# Elimination of aberrantly specified cell clones is independent of interfacial Myosin II accumulation

**DOI:** 10.1242/jcs.259935

**Published:** 2023-07-11

**Authors:** Olga Klipa, Menna El Gammal, Fisun Hamaratoglu

**Affiliations:** Cardiff University, School of Biosciences, Cardiff, CF10 3AX, UK

**Keywords:** *Drosophila*, Myosin, Apterous, Cell elimination, Homeostasis, Tension

## Abstract

Spatial organization within an organ is essential and needs to be maintained during development. This is largely implemented via compartment boundaries that serve as barriers between distinct cell types. Biased accumulation of junctional non-muscle Myosin II along the interface between differently fated groups of cells contributes to boundary integrity and maintains its shape via increased tension. Here, using the *Drosophila* wing imaginal disc, we tested whether interfacial tension driven by accumulation of Myosin is responsible for the elimination of aberrantly specified cells that would otherwise compromise compartment organization. To this end, we genetically reduced Myosin II levels in three different patterns: in both wild-type and misspecified cells, only in misspecified cells, and specifically at the interface between wild-type and aberrantly specified cells. We found that the recognition and elimination of aberrantly specified cells do not strictly rely on tensile forces driven by interfacial Myosin cables. Moreover, apical constriction of misspecified cells and their separation from wild-type neighbours occurred even when Myosin levels were greatly reduced. Thus, we conclude that the forces that drive elimination of aberrantly specified cells are largely independent of Myosin II accumulation.

## Teaser

**Summary:** Myosin II accumulation along the boundary between differently fated cells is not necessary to drive elimination and sorting of harmful cells in *Drosophila* larval wing discs.

## INTRODUCTION

Spatial organization of differently fated cells is central to the development of multicellular organisms. Correctly organized cells are vital in ensuring the formation of fully functional body structures and organs. Thus, cells with inappropriate positional identity, which occasionally arise owing to sporadic mutations or chromatin defects, are detrimental for the whole organism. Fortunately, such cells can be detected and effectively removed from the tissue (Adachi-Yamada et al., 1999; Adachi-Yamada and O'Connor, 2002; Bielmeier et al., 2016; Klipa and Hamaratoglu, 2019). However, the mechanism by which this happens is largely unknown. An ideal model to address this question is the *Drosophila* wing imaginal disc, because of its relatively simple and well-described patterning events and its readily available mosaic techniques (Beira and Paro, 2016).

In *Drosophila*, wing disc cells are organized into four compartments [anterior (A), posterior (P), dorsal (D) and ventral (V)] separated by lineage boundaries. The cellular identity of each compartment is defined by the restricted expression of selector genes. Expression of *engrailed* (*en*) and *invected* (*inv*) grants cells a posterior identity, whereas their absence marks cells for an anterior fate (Morata and Lawrence, 1975; Guillén et al., 1995; Sanicola et al., 1995; Simmonds et al., 1995; Tabata et al., 1995). Likewise, *apterous* (*ap*) expression defines dorsal cells, whereas its absence specifies ventral cells (Diaz-Benjumea and Cohen, 1993; Williams et al., 1993; Blair et al., 1994). The interactions between anterior and posterior cells as well as those between dorsal and ventral cells firstly lead to local activation of short-range signalling molecules and secondly to secretion of the long-range morphogens Decapentaplegic (Dpp) and Wingless (Wg) along anteroposterior (A/P) and dorsoventral (D/V) boundaries, respectively (Basler and Struhl, 1994; Zecca et al., 1995; Diaz-Benjumea and Cohen, 1995; Couso et al., 1995; Rulifson and Blair, 1995). Therefore, the compartment boundaries act both as fences between different cell populations, preventing their mixing, and as organizing centres for further patterning events (Ruiz-Losada et al., 2018; Wang and Dahmann, 2020).

Conceptually, two mechanical properties keep cell populations apart: differences in cohesive strength between interacting cell types (differential adhesion) and local increase of junction tension along the interface (interfacial tension) (Steinberg, 1963; Foty and Steinberg, 2005; Tepass et al., 2002; Brodland, 2002; Lecuit and Lenne, 2007; Fagotto, 2014; Wang and Dahmann, 2020). The ways in which differential adhesion and interfacial tension are genetically encoded and controlled by upstream signalling events have been the subject of intense research. In the case of the D/V boundary, both processes contribute to boundary formation and maintenance. The Apterous (Ap) target genes *capricious* (*caps*), *tartan* (*trn*), *fringe* (*fng*), *Serrate* (*Ser*), *Delta* and *bantam*-microRNA (*mir-ban*) were shown to play key roles in the process (Milán et al., 2001; Milán and Cohen, 2003; Rauskolb et al., 1999; O'Keefe and Thomas, 2001; Becam et al., 2011). The transmembrane proteins Capricious and Tartan are dorsally expressed at the time of D/V boundary formation and contribute to cell segregation (Milán et al., 2001). They are thought to function as ligands to a yet-to-be-discovered dorsal specific receptor (Milán et al., 2005). Thus, the initial separation of dorsal and ventral cells is achieved via early dorsal expression of Capricious and Tartan (Milán and Cohen, 2003). Once the boundary is formed, its maintenance depends on Notch (N) activity (Rauskolb et al., 1999; Micchelli and Blair, 1999; Milán and Cohen, 2000, 2003; Major and Irvine, 2005; Becam et al., 2011; Michel et al., 2016). Ap induces the expressions of the N ligand Serrate and its modulator Fringe in dorsal cells and restricts Delta expression to the ventral cells, ensuring a stripe of N signalling along the D/V boundary (Rauskolb et al., 1999; O'Keefe and Thomas, 2001; Becam and Milán, 2008). This stripe of N ensures boundary maintenance via reduced proliferation at the D/V boundary by repression of bantam (Becam et al., 2011), as well as by increasing the cell bond tension as a result of accumulations of Myosin II and Enabled (Ena), an actin regulator (Michel et al., 2016; Becam et al., 2011). It was shown that actomyosin filaments are enriched along the D/V boundary (Major and Irvine, 2005, 2006) and cortical tension is higher at the boundary than elsewhere in the compartments (Aliee et al., 2012; Umetsu et al., 2014). Actomyosin enrichment and elevated tension at lineage boundaries ensure that differently fated cells are kept separate and straight boundaries are maintained (Landsberg et al., 2009; Monier et al., 2010; Aliee et al., 2012; Umetsu et al., 2014; Rudolf et al., 2015).

Crucially, if clones of cells mutant for a selector gene arise in a compartment in which that gene is expressed, the clones are either relocated to the opposite compartment or eliminated from the tissue completely (Morata and Lawrence, 1975; Diaz-Benjumea and Cohen, 1993; Blair et al., 1994; Milán et al., 2002; Klipa and Hamaratoglu, 2019). Cell sorting (relocation) and elimination were also reported for clones that ectopically express a range of other fate-specifying transcription factors [including Cubitus interruptus (Ci), Vestigial (Vg), Homothorax (Hth), the Iroquois complex, Spalt (Salm) and Optomotor-blind (Omb, also known as Bi)] (Dahmann and Basler, 2000; Baena-Lopez and García-Bellido, 2006; Villa-Cuesta et al., 2007; Shen et al., 2010; Bielmeier et al., 2016). Similarly, cell clones with abnormal levels of Wg or Dpp signalling were shown to form cysts or undergo apoptosis (Adachi-Yamada and O'Connor, 2002; Moreno et al., 2002; Giraldez and Cohen, 2003; Johnston and Sanders, 2003; Gibson and Perrimon, 2005; Shen and Dahmann, 2005; Widmann and Dahmann, 2009). Altogether, these numerous observations demonstrate the ability of a tissue to identify and remove, or sort out misspecified cells. However, the mechanism by which this is achieved remains unclear.

**Fig. 1.**
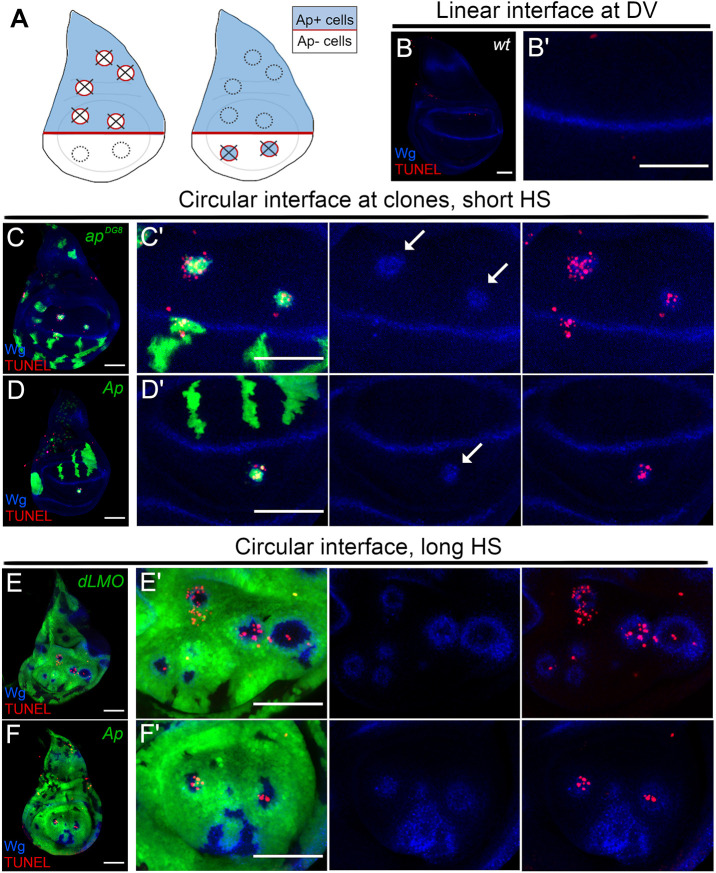
**The majority population determines the correct cell fate in the wing disc, and the cells of ‘wrong’ identity undergo apoptosis.** (A) Schematics of third instar wing discs. Ap (blue) is expressed in the dorsal compartment and Wg (red) expression marks the D/V boundary. Removal of Ap function from the dorsal compartment (left) or ectopic Ap expression in the ventral compartment (right) leads to Wg expression at the interface of Ap^+^ and Ap^−^ cells and eventual elimination (crosses). (B,B′) Third instar wing disc (B) and its pouch region (B′) showing Wg antibody staining in blue and TUNEL staining in red. Wg expression at the boundary reveals a straight interface between Ap-expressing dorsal cells and ventral cells without Ap. (C–D′) Wing discs bearing GFP-expressing (green) clones that are mutant for *ap^DG8^* (C) or ectopically expressing Ap (D) and their pouch regions (C′,D′). All panels show Wg antibody staining in blue, and TUNEL in red. Arrows point to circular ectopic boundaries associated with misspecified clones that are undergoing elimination. (E,F) A long heat shock (HS) generates discs that are almost entirely composed of GFP-positive (green) *dLMO* (E) or Ap-expressing (F) cells. (E′,F′) Pouch regions of the discs shown in E,F with separate channels showing Wg (blue) and TUNEL (red) stainings. Wild-type islets trapped between overexpressing cells undergo apoptosis. Dorsal is up, anterior is to the left in all panels. Images are representative of a minimum of 15 discs examined in three independent experiments. All scale bars: 50 μm.

In this study, we focused on cells with aberrant dorsoventral identity. Cells that lack Ap activity are eliminated from the dorsal compartment of the wing disc, whereas cells that ectopically express Ap are removed from the ventral part (Fig. 1A) (Milán et al., 2002; Klipa and Hamaratoglu, 2019). There are at least three mechanisms involved in the clearance: relocation to the identity-appropriate compartment, apoptosis and basal extrusion (Klipa and Hamaratoglu, 2019). Interaction of *ap*-expressing and *ap*-non-expressing cells at the boundary of misspecified clones induces ectopic activation of N and Wg signalling. The clone boundaries become smooth, suggesting their separation from the surrounding wild-type (wt) cells (Milán and Cohen, 2003). This greatly resembles the process at the D/V boundary. Altogether, these observations raise the question of whether the events involved in the formation of compartment boundaries during normal development also play a central role in cell elimination. Accordingly, modulation of N signalling or reestablishment of adhesive properties allows aberrantly specified cells to remain in the tissue (Milán et al., 2002; Milán and Cohen, 2003). Moreover, it was reported that Myosin II accumulation and increased tension at the D/V boundary depends on N activity (Major and Irvine, 2006; Michel et al., 2016). Thus, separation of cells with incorrect dorsoventral identity and their subsequent elimination could be mediated by increased tension, driven by actomyosin contractility along clone borders. Importantly, this was proposed to be a general mechanism by which the tissue identifies and responds to the presence of misspecified cells (Bielmeier et al., 2016). Indeed, Bielmeier and colleagues reported accumulation of actomyosin filaments at the interface between differently fated cells upon misexpression of a range of fate-specifying genes (Bielmeier et al., 2016). The beauty of this model is in that it can account for the elimination of any misspecified clone from the tissue regardless of the genetic identity of the clone, and the particular events that culminates in actomyosin accumulation. Notably, when considering segregation of clonal populations from their neighbours, it is also important to consider potential cell-junction tension changes in the bulk of the clone, which were shown to contribute to sorting along with changes at the clonal boundary (Bosveld et al., 2016).

Here, using the *Drosophila* wing imaginal disc, we examined the role of non-muscle Myosin II, the main regulator of contractile forces in a cell, in elimination of cell clones with inappropriate dorsoventral identity. To address this question, we interfered with Myosin II activity in three different patterns: in the whole disc, inside the clone and at the clone boundary. We then examined the topology and elimination efficiency of the clones. Surprisingly, we found that both clone elimination and their separation from the surrounding cells do not rely on Myosin II accumulation at the clonal boundary.

## RESULTS

### Apposition of differently fated cells induces apoptosis when the contact interface is circular but not when it is linear, ensuring the removal of the underrepresented cell population

Both dorsal and ventral cell populations are healthy and viable on their own (Fig. 1B). The interaction of Ap-expressing cells (dorsal identity) and Ap-non-expressing cells (ventral identity) causes activation of boundary signalling (N and Wg) (Rauskolb et al., 1999). However, the outcome of this event at the D/V compartment boundary is different to that around mispositioned clones (Fig. 1B–D). The activation of N and Wg signalling along the compartment boundary does not lead to cell death (Fig. 1B,B′). It is a physiological condition that is necessary for proper patterning. In contrast, the interaction of misspecified cell clones with their wt neighbours induced apoptosis, as revealed by the terminal deoxynucleotidyl transferase dUTP nick end labelling (TUNEL) assay. Both *ap* mutant (*ap^DG8^*) cells in the dorsal compartment and Ap-expressing cells in the ventral compartment showed strong induction of apoptosis (Fig. 1C–D′, arrows). Thus, the apoptosis observed purely relies on cell–cell interactions and does not depend on cell identity. To further test this idea, cell clones expressing either *dLMO* (negative regulator of Ap activity; also known as *Bx*) (Milán et al., 1998) or *UAS-ap* were induced using a long heat-shock scheme to allow these cells to occupy almost the whole wing disc. The remaining wt cells, i.e. cells that were GFP negative, were highly underrepresented (Fig. 1E–F′). The wing discs with such clones did not have regular D/V boundaries, as revealed by Wg staining. Instead, they contained boundary signals in circles that were formed around the wt islands (Fig. 1E–F′). Importantly, the interaction of differently fated cells in this scenario also resulted in cell death. However, in this case, apoptosis was preferably associated with the wt cell population (Fig. 1E–F′). This suggests that among two differently fated cell populations that interact with each other, the underrepresented one is designated for elimination, regardless of its identity. When one cell population is underrepresented, the boundary it forms with its surrounding neighbours is circular. In contrast, the compartment boundary, which is formed between relatively big and similarly sized cell populations, is linear. The former is associated with apoptosis and elimination, whereas no cell death is induced in the latter. Thus, it is possible that the presence of relatively small groups of cells that disrupt global patterns (misspecified cells) could be detected by a circular interface. The underlying mechanism as to how interface shape determines the output could be attributed to the Laplace pressure and interface contractility. The Laplace pressure, which refers to the pressure difference between the inside and the outside of a curved boundary, is inversely proportional to the radius of the boundary. Therefore, a smaller clone is under higher Laplace pressure exerted by its boundary. According to this model, increased contractility around relatively small cell clusters causes irresistible mechanical stress (compression and apical constriction) in these encircled groups of cells, leading to induction of apoptosis and subsequent elimination (Bielmeier et al., 2016).

### Evidence for interface contractility between Ap-positive and Ap-negative cells

**Fig. 2.**
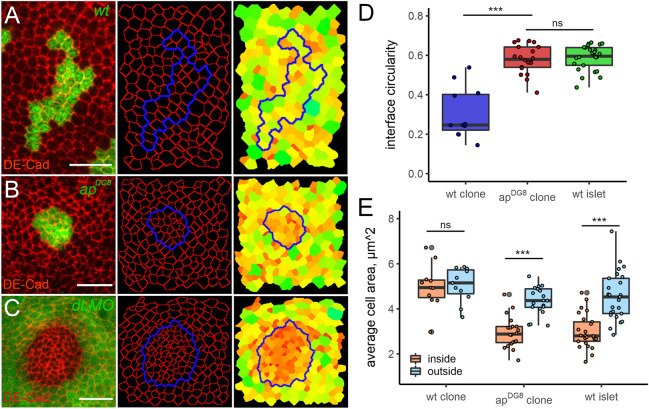
**Cells undergo apical constriction within the patches of minority identity.** (A–C) Representative examples of GFP-marked (green) wt (A) or *ap^DG8^* mutant (B) clones, and a GFP-negative wt islet in a *dLMO*-expressing disc (C). DE-Cad (red) staining was used to reveal the apical cell outlines in Epitools. Clone boundaries are shown in blue. The right panels show heatmaps of apical areas, from smallest (0.2 µm^2^, dark red) to largest (12 µm^2^, light green). Scale bars: 10 μm. (D,E) Quantifications of interface circularity (D) and average cell area (E) inside (orange) and outside (blue) 11 wt, 20 *ap^DG8^* clones and 25 wt islets trapped in *dLMO*-expressing discs. Boxes represent the 25–75th percentiles, whiskers show the range of the data with extremes not exceeding 1.5× the interquartile range from the middle 50% of the data. The data points outside this range are defined as outliers and are indicated by the grey points. The median is marked with a line. ns, not significant; ***P<0.001.

**Fig. 3.**
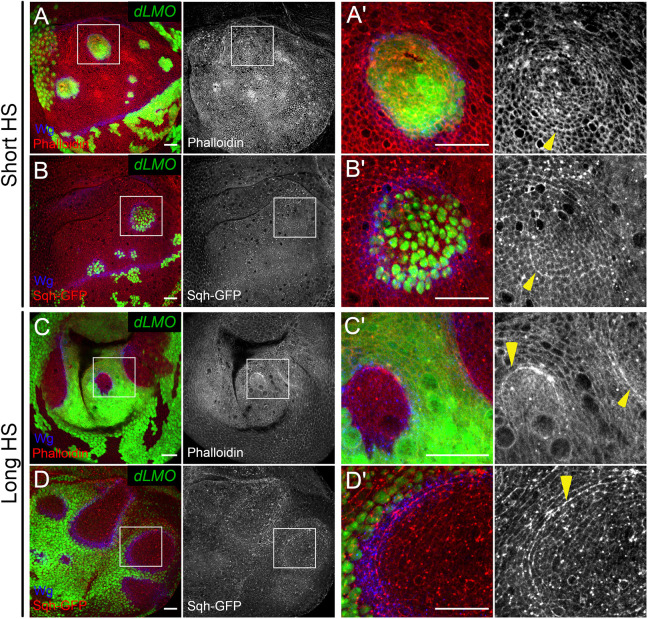
**The actomyosin network is enriched at the interface between cell populations of different identities.** (A–B′) Wing discs with *dLMO*-expressing cells (green), stained for Wg (blue) and Phalloidin (red, grey) (A,A′) or carrying the *Sqh-GFP* transgene (red, grey) (B,B′). (C–D′) Wing discs subjected to long heat shock generating wt islets (non-green regions) surrounded by *dLMO*-expressing cells (green). Wg is shown in blue; red in overlay or single grey channels show Phalloidin (C,C′) or Sqh–GFP (D,D′). A′–D′ show the zoomed-in versions of the white boxes in A–D. Arrowheads (yellow) point to interfaces. Dorsal is up, anterior is to the left in all panels. Images are representative of a minimum of 15 discs from three independent experiments. All scale bars: 20 μm.

To investigate whether the interface between differently fated cell populations in our scenario had elevated tension, we analysed the circularity of the interfaces at wt GFP-marked clones surrounded by wt cells (Fig. 2A), *ap* mutant clones located in the dorsal compartment (Fig. 2B) and Ap-positive wt islands surrounded by *dLMO*-expressing cells (Fig. 2C). The interface between differently fated cells was more circular compared to the interface between cells of the same identity (Fig. 2D). To test whether the enclosed cells outlined by highly circular boundaries experienced compression, we measured the apical areas of individual cells from both sides of the interface, within the clones and outside. The cell membranes were revealed by DE-Cadherin (or DE-Cad, also known as Shg) staining and the analysis was done using Epitools (Heller et al., 2016). The cells of wt clones had similar apical areas as the surrounding wt cells (Fig. 2A, heatmap; Fig. 2E). In contrast, the apical areas of the *ap^DG8^* cells and those of cells in wt islands were much smaller compared to those of the surrounding cells (Fig. 2B,C, heatmaps; Fig. 2E). Importantly, the surrounding cells had comparable apical areas in all three scenarios (Fig. 2E, blue bars). Such apical constriction of underrepresented cells could be easily explained by interface contractility. Traditionally, contractility and high tension are associated with enrichment of actomyosin filaments. Therefore, we analysed F-actin (using Phalloidin staining) and non-muscle Myosin II (using a *sqh-GFP* reporter) distribution at misspecified cell clones and wt islets surrounded by cells of different identity. Both F-actin and Myosin II accumulated at adherens junctions along the borders of many (but not all) *dLMO* clones that remained in the dorsal compartment (Fig. 3A–B′). Interestingly, in some cases, elevated levels of Myosin were observed not only along the interface junctions, but also at the junctions inside the misspecified clones, which might contribute to apical constrictions (Fig. 7A,A′). The actomyosin enrichment was even more prominent along the interfaces between wt islands and *dLMO*-expressing cells generated by a long heat shock. Very thick F-actin and Myosin cables were observed in nearly all cases (Fig. 3C–D′). Altogether, these observations provide evidence of increased contractility along the interface and are in favour of the mechanical stress model. According to this model, the elimination of misspecified cells is due to their deformation mediated by actomyosin accumulation and increased contractility at the interface. If this is the case, then depletion of Myosin would be sufficient to abolish the elimination. In order to test this hypothesis, we designed experiments in which the level of Myosin was reduced in the whole disc, within the misspecified clones and specifically at the clone boundary.

### Misspecified clones induced in *zipper* mutant wing discs are still eliminated

**Fig. 4.**
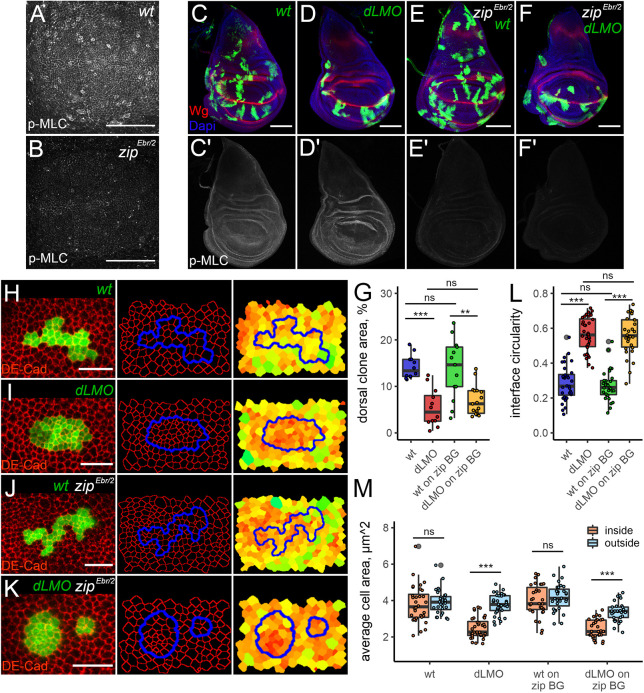
**A reduction in Myosin activity has no effect on interface circularity, apical constriction and *dLMO*-expressing cell clone elimination.** (A,B) Phospho-Myosin light chain (p-MLC) staining (grey) was reduced in the *zip^Ebr/2^* mutant background (B) compared to that in wt (A). For quantification, see Fig. S1A. The samples were processed in parallel. Images are representative of a minimum of 12 discs. (C–F′) Wing discs containing control (C,E) or *dLMO*-expressing (D,F) clones in a wt (C,D) or *zip^Ebr/2^* mutant background (E,F). C′–F′ show p-MLC staining (grey) alone. Dorsal is up, anterior is to the left in all panels. Images are representative of a minimum of 12 discs. (G–K) Representative examples of GFP-marked (green) wt (H,J) or *dLMO*-expressing (I,K) clones in a wt (H,I) or *zip^Ebr/2^* mutant background (J,K). DE-Cad (red) staining was used to reveal the apical cell outlines in Epitools. Clone boundaries are shown in blue. The right panels show heatmaps of apical areas, from small (red) to large (green). Quantification of dorsal clone area in the indicated genotypes is shown in G. A minimum of 12 discs were analysed per genotype. (L,M) Quantifications of interface circularity (L) and average cell area (M) inside (orange) and outside (blue) a minimum of 30 clones for each genotype. Box plots are presented as described in Fig. 2. Scale bars: 50 μm (A,B); 100 μm (C–F); 10 μm (H–K). ns, not significant; **P<0.01; ***P<0.001.

Non-muscle Myosin II is a vital molecule and its function is highly important for development. That is why animals bearing a strong Myosin mutation are not viable. However, some combinations of hypomorphic alleles of Myosin heavy chain *zipper* (*zip*) are less harmful and flies can survive until later stages. It was shown that *zip^Ebr^/zip^2^* is the strongest combination that allows recovery of third instar larvae (Major and Irvine, 2006). We assessed Myosin levels in *zip^Ebr^/zip^2^* wing discs using antibodies against the phosphorylated form of Myosin light chain (p-MLC), which is an indicator of the assembled active form of the Myosin complex. In *zip^Ebr^/zip^2^* wing discs, Myosin levels were greatly reduced; residual p-MLC levels did not exceed 25–30% of the levels seen in wt discs (Fig. 4A,B; Fig. S1A). Interestingly, the mutant discs were similar in size to wt discs of the same developmental stage (Fig. 4C,E). To evaluate the elimination efficiency of misspecified clones in a Myosin-depleted background, we generated GFP-labelled wt and *dLMO*-expressing clones in both wt and *zip^Ebr^/zip^2^* imaginal discs at 58 h after egg laying (AEL) and examined clone recovery in the dorsal compartment 60 h later (day 5 AEL). As expected, *dLMO* clones were highly underrepresented in the dorsal part of the disc compared to wt clones (Fig. 4C,D,G). The areas of dorsal compartment occupied by wt clones in the wt and *zip^Ebr^/zip^2^* backgrounds were comparable (Fig. 4C,E,G). The recovery rate of *dLMO* clones generated in *zip* mutant discs was not significantly different from that of *dLMO* clones generated in wt discs. In both cases, misspecified clones were eliminated efficiently (Fig. 4D,F,G). Next, we examined whether the *zip^Ebr^/zip^2^* background affects the increased interface circularity and apical constriction associated with the aberrantly specified clones. Surprisingly, we found that dorsal *dLMO* clones in *zipper* mutant discs had very smooth borders (Fig. 4K) and their circularity did not differ from that of dorsal *dLMO* clones generated in wt discs (Fig. 4I,K,L). Moreover, similar to *dLMO*-expressing cells induced in wt discs (Fig. 4I), *dLMO*-expressing cells in *zip* mutant discs (Fig. 4K) had smaller apical areas compared to those of the surrounding cells (Fig. 4M). As expected, the apical cell areas of GFP-labelled clones in either wt (Fig. 4H) or *zip* mutant (Fig. 4J) backgrounds were not distinct from their surrounding neighbours (Fig. 4M). Thus, we conclude that reduction of Myosin using a *zip^Ebr^/zip^2^* allelic combination does not result in a relaxation of tension along the borders of misspecified clones, nor does it prevent their elimination.

### Myosin II depletion inside the misspecified clones has a mild effect on clone recovery

**Fig. 5.**
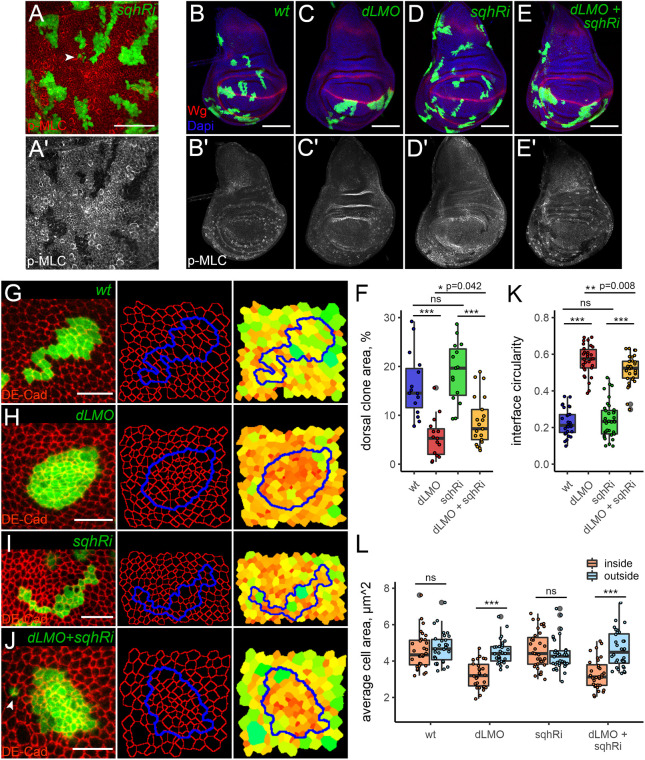
**Knocking down Myosin light chain within *dLMO*-expressing clones mildly influences clone recovery and circularity.** (A,A′) Phospho-Myosin light chain (p-MLC) staining (red in A and grey in A′) was greatly reduced in *sqh-RNAi* (*sqhRi*)-expressing cell clones (green). The arrowhead in A points to dispersed cells. Images are representative of 15 discs. (B–E′) Wing discs containing control (B), *dLMO*-expressing (C), *sqhRi*-expressing (D) or *dLMO-* and *sqhRi*-expressing clones (E). B′–E′ show p-MLC staining (grey) alone. Dorsal is up, anterior is to the left in all panels. Images are representative of a minimum of 15 different discs per genotype. (F–J) Representative examples of GFP-marked (green) wt (G), *dLMO*-expressing (H), *sqhRi*-expressing (I) or *dLMO-* and *sqhRi*-expressing (J) clones. DE-Cad (red) staining was used to reveal the apical cell outlines in Epitools. Clone boundaries are shown in blue. The right panels show heatmaps of apical areas, from small (red) to large (green). The arrowhead in J indicates a cell dissociated from a clone. Quantification of dorsal clone area in the indicated genotypes is shown in F. A minimum of 15 discs were analysed per genotype. (K,L) Quantifications of interface circularity (K) and average cell area (L) inside (orange) and outside (blue) a minimum of 30 clones for each genotype. Box plots are presented as described in Fig. 2. Scale bars: 50 μm (A); 100 μm (B–E); 10 μm (G–J). ns, not significant; *P<0.05; **P<0.01; ***P<0.001.

Next, we obstructed Myosin II function within misspecified clones by knocking down the regulatory light chain of Myosin II, *spaghetti squash* (*sqh*). The *UAS-sqh-RNAi (sqhRi)* line used is very effective in depleting Myosin as revealed by phospho-Myosin staining. The Myosin levels in cell clones expressing *sqhRi* did not exceed 20% of normal levels, with no residual Myosin cables detected at the junctions of these cells (Fig. 5A,A′; Fig. S1B). Interestingly, many *sqhRi* clones were dispersed in appearance, where some clonal cells disengaged from their siblings and became fully surrounded by wt cells (Fig. 5A, arrowhead; Fig. S2A,A′). To analyse this effect quantitatively, we introduced two parameters: the mixing index, defined as the number of wt cells in direct contact with one clonal cell at the frontline (Fig. S2B,B′), and the contact length, defined as the clone perimeter divided by the number of clonal cells at the frontline (Fig. S2B″). Both the mixing index and the contact length of *sqhRi* clones were significantly higher than those of wt clones (Fig. S2C,D). Thus, *sqhRi* impairs clone integrity and allows clonal cells to split apart by cell divisions and rearrangements more easily. These observations suggest that knocking down Myosin II by *sqhRi* compromises tensile forces at cell junctions. To assess the effect of *sqhRi* on elimination of misspecified clones, wt, *dLMO*, *sqhRi* and *dLMO+sqhRi* clones were induced at 58 h AEL and clone recovery in the dorsal disc was measured 60 h later (day 5 AEL). The *dLMO*-expressing clones were eliminated as expected (Fig. 5B,C,F) and the recovery of *sqhRi* cells was comparable to that of wt cells (Fig. 5D,F). Interestingly, co-expression of *dLMO* with *sqhRi* also resulted in efficient clone elimination from the dorsal compartment (Fig. 5E,F). However, the recovery rate of these clones was slightly higher compared to that of clones expressing *dLMO* alone (Fig. 5F). Thus, *sqh* depletion within the clone provides a mild rescue effect. Next, we examined how *sqhRi* co-expression affects tension along misspecified clone boundaries. As mentioned above, *sqhRi*-expressing clones easily split apart and have a high mixing index and high contact lengths (Fig. S2). However, this increased mixing tendency of *sqhRi* cells was not reflected in our measurements of interface circularity; *sqhRi* clones did not differ from wt clones in terms of circularity (Fig. 5G,I,K). This is likely owing to the fact that we only analysed clones consisting of ≥10 cells for their circularity. If all the dispersed cells could be included in our analysis as part of the bigger clone they separated from, lower circularity would be yielded for *sqhRi*-expressing clones. As expected, *dLMO* clones rounded up efficiently and had very smooth borders (Fig. 5H,K). Clones co-expressing *dLMO* and *sqhRi* were significantly less circular compared to those expressing *dLMO* alone (Fig. 5H,J,K). Moreover, in some instances, *dLMO+sqhRi* clonal cells appeared to be detached from the main clone (Fig. 5J, arrowhead). Nevertheless *dLMO+sqhRi* clone borders were much smoother than those of either wt or *sqhRi* clones (Fig. 5G,I–K). In addition, *sqhRi* co-expression did not change the mixing index or the contact length of misspecified clones (Fig. S2C,D). Notably, the *sqhRi* clones were highly heterogeneous in cell size, hosting both very small and very big cells (Fig. 5I, heatmap). However, the average apical areas of *sqhRi*-expressing cells were similar to those of the surrounding wt cells (Fig. 5I, heatmap; Fig. 5L). Strikingly, the apical areas of cells within *dLMO* as well as *dLMO+sqhRi* clones were much smaller than those of surrounding cells (Fig. 5H,J, heatmaps; Fig. 5L). Thus, co-expression of *sqhRi* did not prevent the apical constriction of misspecified cells. Finally, we pursued an alternative approach to interfere with Myosin activity and used a UAS-RNAi against Rho kinase (Rok) (*rokRi*), which phosphorylates and activates Myosin II (Mizuno et al., 1999). Cell clones expressing *rokRi* behaved very similar to *sqhRi* clones (Fig. S3D). Importantly, our control experiments performed in parallel behaved as expected (Fig. S3A–C,F). Co-expression of *rokRi* along with *dLMO* had no effect on clone recovery (Fig. S3E,F). We conclude that although depletion of Myosin II light chain in misspecified clones led to interface relaxation and slightly increased clone recovery, the effect was too mild to grant Myosin a central role in the elimination of misspecified cells.

### No evidence that Myosin II depletion along boundaries of misspecified clones prevents their elimination

**Fig. 6.**
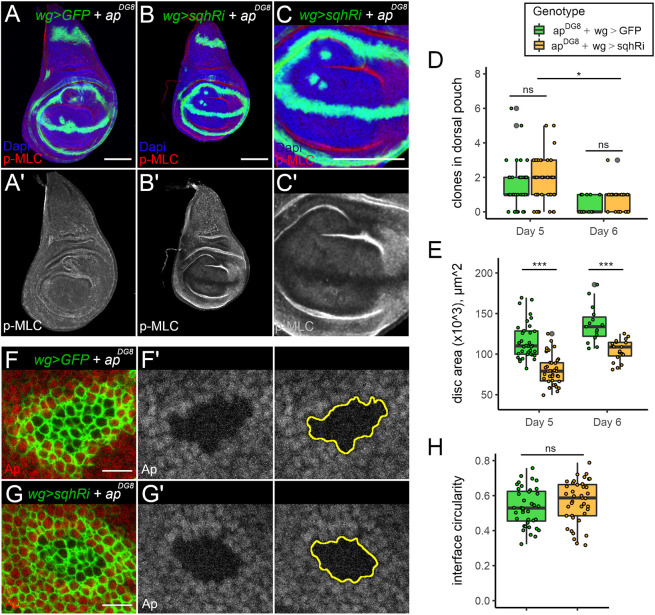
**Knocking down Myosin light chain around *ap^DG8^* clones does not affect clone recovery and circularity.** (A–C′) Day 5 wing discs bearing unmarked clones that are mutant for *ap^DG8^* and expressing GFP (A) or GFP and *sqhRi* (B,C′) under *wg::Gal4* control. The remaining clones in the pouch are detectable with the ectopic *wg::Gal4*-driven *GFP* induced around them. Panel C shows the pouch region of the disc in panel B. Nuclei are marked with DAPI (blue) and p-MLC staining is shown in red (top panels) or in grey (lower panels). Dorsal is up, anterior is to the left in all panels. (D,E) Quantification of the number of remaining clones in the dorsal pouch (D) and disc areas (E) in the indicated genotypes. At least 36 discs per genotype for day 5 and at least 15 discs per genotype for day 6 were considered for the analysis. Expression of *sqhRi* caused growth delay, and further elimination occurred between the two time points. (F–G′) Representative *ap^DG8^* clones expressing GFP (F) or GFP and *sqhRi* (G) under *wg::Gal4* control. F′,G′ show Ap antibody staining in grey, and the estimated clone boundaries based on this staining are marked in yellow. (H) Quantification of the interface circularity of the remaining *ap^DG8^* clones with (yellow) or without (green) *wg::Gal4*-driven *sqhRi* in the dorsal pouch. Forty clones were measured for each genotype. Box plots are presented as described in Fig. 2. Scale bars: 100 μm (A–C); 10 μm (F,G). ns, not significant; *P<0.05; ***P<0.001.

The approach described above targeted Myosin in clonal cells only, without affecting its expression in flanking wt cells. Arguably, both sides can contribute to Myosin enrichment and tension observed along clone boundaries. We therefore sought to design an experiment in which we reduced Myosin levels from both sides of the clone boundary. We took advantage of the fact that interaction of Ap-positive and Ap-negative cells results in Wg expression in flanking cells from both sides of the boundary. Thus, a *wg::Gal4* construct (Alexandre et al., 2014) was used to drive expression of *sqhRi* at the boundaries of misspecified cells. First, we analysed how the expression of *sqhRi* in the Wg domain affects the D/V boundary of an otherwise wt wing disc (*wg>sqhRi*). The junctional Myosin levels were highly reduced at the D/V boundary of such discs in comparison to those of control discs (w*g>GFP*), as revealed by p-MLC staining at the apical side (Fig. S4A,A′,D,D′). The D/V boundaries of *wg>sqhRi* discs appeared to be narrower than those of *wg>GFP* discs and were occasionally disrupted, as revealed by GFP signals (Fig. S4B–C′,E–F′). Crucially, *wg>sqhRi* discs were smaller than the controls, suggesting some developmental delay (Fig. 6A,B,E, see below). We induced *ap^DG8^* cells and analysed their recovery rates in both *wg>sqhRi* and *wg>GFP* discs. The clones were generated at 58 h AEL and analysed 60 h later (day 5 AEL). The presence of *ap* mutant clones in the dorsal discs was revealed by the ectopic GFP expression induced around the clones (Fig. 6A–C). The number of remaining misspecified clones in the dorsal pouch of *wg>sqhRi* discs was slightly, but not significantly, higher than that in the dorsal pouch of *wg>GFP* discs. On average, two misspecified clones per dorsal pouch remained in *wg>sqhRi* discs (Fig. 6A,B,D). However, *wg>sqhRi* larvae experienced some developmental delay, resulting in smaller discs at the specified time point (Fig. 6E). To confirm that *ap* mutant clones underwent elimination in these discs, we also dissected larvae on day 6 AEL (84 h after heat shock). We found that, on day 6, almost no misspecified clones remained in the dorsal pouch of *wg>sqhRi* discs (Fig. 6D). This suggests that, despite the reduction of Myosin II along the clone boundary, the elimination of misspecified clones still takes place. Next, using Ap staining to define clone outlines, we analysed the circularity of misspecified clones. Note that Ap, being a transcription factor, predominantly localizes to the nucleus. Therefore, we were unable to analyse clone circularity at the level of the adherens junctions (as in previous cases); instead, we measured it more basally. To ensure that more misspecified clones were retained for analysis, we generated the clones slightly later, at 68 h AEL, and examined them 50 h later (118 h AEL). We noted that *wg::Gal4* drives expression of GFP in flanking cells as far as two to three cells on either side of the interface (Fig. 6F,G). In most cases, owing to the small size of the misspecified clones, all clonal cells appeared to be GFP-positive. Surprisingly, the clones in the control (*wg>GFP*) and the experimental (*wg>sqhRi*) discs were both round, with very similar circularity (Fig. 6F′,G′,H). Altogether, these data do not support the model in which the central role of clearance of aberrantly specified clones is attributed to Myosin-driven contractility at the clone interface.

## DISCUSSION

The forces produced by actomyosin cables are essential and were implicated in many biological processes, such as tissue closure, wound healing, tissue extension, tube formation, compartment organization and cell elimination (Röper, 2013; Bielmeier et al., 2016; Wang and Dahmann, 2020). The importance of actomyosin cables in some of these processes has been recently revised (Pasakarnis et al., 2016; Ochoa-Espinosa et al., 2017). In this study, we challenge the model that attributes a central role for interface contractility mediated by actomyosin filaments in the identification and elimination of aberrantly specified cells.

The interactions of differently fated cells form boundaries that restrict cell mixing and maintain tissue organization. Previous work has shown that accumulation of actomyosin filaments and increased cell bound tension are vital to boundary function (Major and Irvine, 2006; Landsberg et al., 2009; Aliee et al., 2012; Monier et al., 2010). Although this physiological mechanism works well in separating two similarly sized cell populations (such as two compartments), it could be problematic when the populations differ in size and one encloses the other. The cells of the underrepresented group encircled by a highly tense boundary experience compression (Bielmeier et al., 2016). Mechanical cell deformation can, in turn, induce cell death and elimination, possibly via downregulation of the EGFR/ERK signalling pathway (Moreno et al., 2019). This model elegantly explains how misspecified cells are detected and why apoptosis is associated with misspecified cells, but not with compartment boundaries.

In our study, we used cells with aberrant dorsoventral identity to test this model. We demonstrated that the interface between Ap-positive and Ap-negative cell groups was indeed enriched for both F-actin and Myosin II cables (Fig. 3). The interface was highly smooth and round (Fig. 2A–D), suggesting increased tension. Moreover, the cells of underrepresented populations show obvious apical constrictions (Fig. 2E). All these observations are in favour of the model and are consistent with previous studies (Bielmeier et al., 2016; Major and Irvine, 2005, 2006). However, when we tested the involvement of non-muscle Myosin II in the process, we found that it was largely dispensable.

**Fig. 7.**
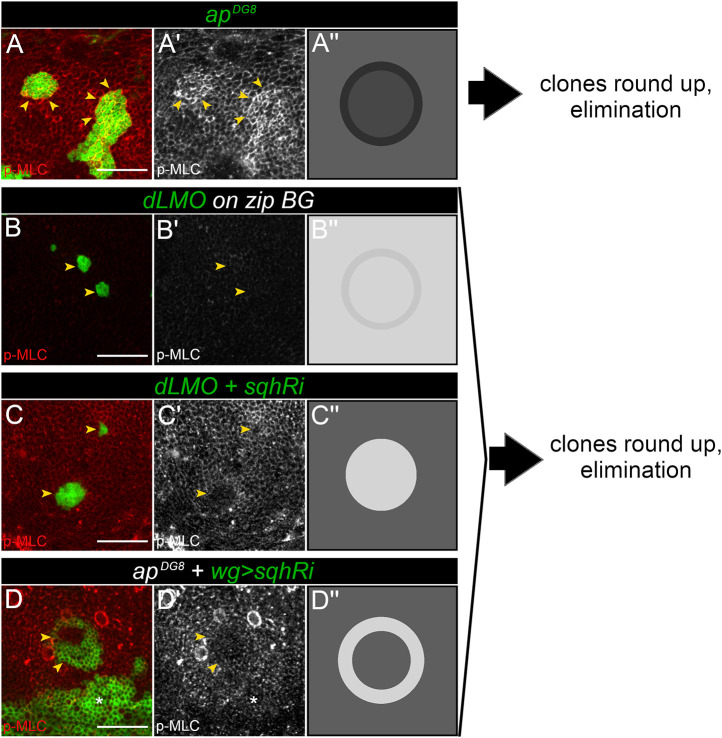
**Interfering with Myosin function with different approaches is not sufficient to abolish elimination of the misspecified cell clones.** (A–D) Representative images (A–D′) and schematics (A″–D″) to summarize and compile the data presented in Fig. 3 (A), Fig. 4 (B), Fig. 5 (C), and Fig. 6 (D). A GFP-marked *ap^DG8^* clone (A), a clone expressing *dLMO* and GFP (green) in a *zip^Ebr/2^* mutant background (B), a clone expressing *dLMO* and *sqhRi* (green) (C) and an *ap^DG8^* clone with *wg::Gal4*-driven *sqhRi* (green) (D) are shown. Wg induction around the clone (D) was used to downregulate Myosin function at the clone boundary. The GFP-positive area below the clone (white asterisks) corresponds to the D/V boundary. Panels A–D show clones of corresponding genotype in green and p-MLC staining in red. Panels (A′–D′) show p-MLC staining alone in grey. Yellow arrowheads point to the clone borders. Panels A″–D″ schematize the Myosin II distribution for each approach. Scale bars: 20 μm.

We have assessed the importance of Myosin-driven tension in the elimination of misspecified cell populations using three different approaches: tissue-wide reduction of Myosin II function (Fig. 7B), inhibition in misspecified cells only (Fig. 7C) or inhibition specifically in cells flanking the interface on either side (Fig. 7D). None of the approaches showed a strong rescue effect on misspecified cell clones; moreover, Myosin depletion did not hinder their tendency to round up and form a smooth interface with wt neighbours. These results have several possible explanations. Technical limitations of the approaches used are one possibility. As non-muscle Myosin II is an essential molecule, and its function is required for cytokinesis, it is impossible to remove it completely from cells that are expected to divide and form clones. In our study, a hypomorphic mutant combination of Myosin heavy chain (*zip^Ebr^/zip^2^*) or an RNAi against the Myosin light chain (s*qhRi*) were used to reduce Myosin II levels. Notably, the depletion of Myosin with *zip^Ebr^/zip^2^* was less efficient than that of *sqhRi* and some junctional Myosin was easily detected (Fig. 7B,B′). Therefore, it is possible that the residual Myosin in the *zip^Ebr^/zip^2^* cells could sufficiently generate elevated tension along the interface to cause clone rounding and elimination. Myosin depletion in misspecified cells using *sqhRi* did alter clone circularity and elimination efficiency, but this effect was extremely minor (Fig. 5F,K). Previous work demonstrated that clone separation depends on the relative difference in junctional tension between clone bulk and its boundary, meaning that the reduction of tension within the clonal cells is sufficient to cause clone separation (Bosveld et al., 2016). However, our data show that reduction of tension by *sqhRi* in cell clones did not lead to clone rounding (Fig. 5I,K). Moreover, *sqhRi* clones dissociated and their cells mixed with wt cells more readily (Fig. S2A,C). This is likely because the removal of Myosin from one side of the junction (in clonal cell) cannot be compensated for by Myosin accumulation from the opposite side of the junction (wt cell); thus, the resulting tension of the interfacial junction is also relatively low. However, in the context of misspecified cells, actomyosin levels at interfacial junctions were strongly elevated compared to those in either clone bulk or surrounding wt cells (Figs 3A,B′ and 7A). Hence, if actomyosin accumulation along the shared junction of two contacting cells occurs independently, the reduction of Myosin only in clonal cells could be inadequate in abolishing the increased interfacial tension generated from the wt side. This could explain why the rescue effect in the second approach was too mild. This potential problem was overcome in the third approach, in which Myosin function was depleted in flanking cells from either side of the boundary (Fig. 7D). Although the strategy by which we measured and quantified the efficiency of clone elimination and clone circularity was different from the that of the previous two, the results clearly demonstrated that rounding and elimination of misspecified clones do not rely on Myosin accumulation along the boundary between differently fated cells. Altogether, our results suggest that, in the context of aberrantly specified cell clone elimination, the interfacial contractility is either not as important as it had been previously presumed or the interfacial tension is Myosin independent.

What could be driving the increased tension at the clone boundary and sorting if Myosin II accumulation is not the sole contributor? One possibility is the differential adhesion, which was the first mechanism of cell sorting proposed and has been backed with extensive modelling work as well as *in vitro* experiments (Steinberg, 1963; Foty and Steinberg, 2005; Glazier and Graner, 1993). Recent work showed that differential expression of the transmembrane receptor Toll-1 (Tl) in posterior cells contributes to a straight A/P boundary in the pupal abdominal epithelium in *Drosophila* (Iijima et al., 2020). We observed that some cell clones had increased concentration of DE-Cad, which could contribute to sorting, but this was not a general phenomenon and most of our aberrantly specified cell clones with smooth boundaries did not show differential DE-Cad expression (Fig. S5). Thus, adhesion molecules other than DE-cadherin could play a role in the process. Secondly, there are examples of actin cytoskeleton being reorganized without obvious changes in Myosin II activity in *Drosophila* (Levayer et al., 2015) and cultured cells (Chugh et al., 2017). Hence, we wondered whether actin filaments still accumulated around aberrantly specified cells with reduced Myosin II activity. Similar to the case with DE-Cad levels, actin accumulation was visible around a few clones, but the effect was not consistent. Therefore, we conclude that Myosin II-independent actin accumulation is unlikely to contribute to the elimination of *dLMO*-expressing cell clones. Thirdly, differential localization of atypical myosin Dachs (D) could also generate tension. In the larval wing disc and pupal notum, D is polarized to one side of the cell (Ambegaonkar et al., 2012; Brittle et al., 2012; Bosveld et al., 2012). Bosveld et al. (2012) found that junctions with D enrichment are under twofold higher tension compared to junctions devoid of D in the pupal notum. Notably, Dachs binds to actin filaments, but its motor domain cannot bind to ATP and hydrolyse it (Cao et al., 2014). Hence, how it generates force is unclear. Nevertheless, loss-of-function cell clones of the tumour suppressor gene *fat* (*ft*) round up in a Dachs-dependent manner in the larval wing disc and the pupal notum (Mao et al., 2013; Mao et al., 2006; Bosveld et al., 2016, 2012). It will be important to investigate whether the rounding and clearing of aberrantly specified cell clones depend on Dachs activity.

In conclusion, we revised the model that attributes the main role of identification and elimination of aberrantly specified cells to interfacial supracellular contractility driven by actomyosin cables. The partial rescue effect of *sqhRi* co-expression in misspecified cell clones suggests that Myosin II-mediated tension contributes to cell separation and elimination, but its contribution is rather minor. Thus, we have ruled out a decisive role for Myosin II accumulation-driven tension in the elimination of mispositioned cell clusters.

## MATERIALS AND METHODS

### *Drosophila* strains

The following *Drosophila* stocks were used: *ap^DG8^*, *FRT^f00878^* (Bieli et al., 2015) and *UAS-sqh-GFP* (Royou et al., 2004) (kindly provided by Markus Affolter, Biozentrum, Basel, Switzerland), *UAS-ap* and *UAS-dLMO* (Milán and Cohen, 1999) (kindly provided by Marco Milan, IRB Barcelona, Barcelona, Spain), *zip^Ebr^* (Halsell et al., 2000) and *zip^2^* (Young et al., 1993) (kindly provided by Romain Levayer, Institut Pasteur, Paris, France), *UAS-sqhRNAi* (GD7917) [obtained from the Vienna *Drosophila* Resource Center (VDRC)], *wg::Gal4* (Alexandre et al., 2014) (kindly provided by Jean-Paul Vincent, The Francis Crick Institute, London, UK) and *UAS-rok-RNAi* [BL28797, obtained from the Bloomington *Drosophila* Stock Center (BDSC)]. All crosses were kept on standard medium at 25°C. Flippase expression was induced by a heat shock at 37°C. The detailed fly genotypes and experimental conditions are presented in Table S1.

### Immunohistochemistry

Imaginal discs were prepared and stained using standard procedures. Briefly, larvae were dissected in ice-cold PBS and fixed in 4% paraformaldehyde (PFA) in PBS for 20 min. Washes were performed in PBS containing 0.03% Triton X-100 (PBT) and blocking in PBT containing 2% normal donkey serum (PBTN). Samples were incubated with primary antibodies overnight at 4°C. The primary antibodies used: mouse anti-Wingless [1:2000, deposited to the Developmental Studies Hybridoma Bank (DSHB) by S. M. Cohen, 4D4], rat anti-DE-Cadherin (1:30, deposited to the DSHB by T. Uemura, DCAD2), rabbit anti-phospho-Myosin light chain 2 (Ser19) (1:50, Cell Signaling Technology, 3671) and rabbit anti-Ap (1:1000, described in Bieli et al., 2015). Incubation with secondary antibodies was performed at room temperature for 2 h. The secondary antibodies used were: anti-mouse Alexa Fluor 568 (1:700, Thermo Fisher Scientific, A-11004) and Alexa Fluor 633 (1:700, Thermo Fisher Scientific, A-21235), anti-rat Cy3 (1:300, Jackson ImmunoResearch, 712-165-153) and anti-rabbit Alexa Fluor 568 (1:600, Thermo Fisher Scientific, A-11036) and Alexa Fluor 633 (1:600, Thermo Fisher Scientific, A-21245). Discs were mounted in Vectashield antifade mounting medium with DAPI (Vector Laboratories). For F-actin staining, Phalloidin-tetramethylrhodamine B (Fluka, 77418) was added during incubation with secondary antibodies at a concentration 0.3 μM.

### TUNEL assay

For the TUNEL assay, *In Situ* Cell Death Detection kit, TMR red (Roche) was used. Larvae were dissected in ice-cold PBS and fixed in 4% PFA for 1 h at 4°C. Samples were washed in PBT and blocked in PBTN for 1 h. Next, the samples were incubated with primary antibodies overnight at 4°C and with secondary antibodies for 4 h at 4*°*C. After washing, the tissues were incubated in PBTN overnight at 4°C. Then, samples were permeabilized in 100 mM sodium citrate supplemented with 0.1% Triton X-100 and incubated in 50 μl of TUNEL reaction mix (prepared according to the recipe from the kit) for 2 h at 37°C in the dark. After this step, the samples were washed in PBT for 30 min and mounted in Vectashield antifade mounting medium with DAPI (Vector Laboratories).

### Image acquisition

Image stacks of wing discs were acquired on a Zeiss LSM880 confocal microscope using 20×, 40× and 63× objectives. The pinhole was 1 airy unit. The intervals between *z*-sections were 1 μm thick (for the 20× objective) and 0.4–0.5 μm thick (for the 40× and 63× objectives).

### Image analysis

Clone area measurements were performed using the ImageJ image processing platform. Image stacks were projected using maximum projections. All *z*-slices were included in the projection except for the ones containing signals from the peripodial membrane. The dorsal compartment was selected manually based on Wg staining. For clone detection, Gaussian Blur filter (sigma=2.0) was applied and clones were detected using intensity-based thresholding.

Clone circularity and cell area measurements were performed on images acquired with the 63× objective. Individual clones were selected and processed separately. For experiments presented in Figs 2, 4 and 5, clone perimeter and cell area were measured using the image analysis toolkit Epitools (Heller et al., 2016). Two to eight *z*-slices from the apical part of the stack were projected using selective plane projection (built-in algorithm in Epitools). Cell outlines were detected based on DE-Cad staining. Cell segmentation was performed using the MATLAB-based analysis framework of Epitools. Clones were detected using the GFP signal. Clone area, perimeter and number of clonal cells, as well as cell areas within the clone and in surrounding bulk, were obtained using the CELL_CLONE and CELL_AREA Epitools modules (cellGraph v0.9.1.0), respectively. The circularity was quantified using formula 4π×area/perimeter^2^. The heatmaps of cell areas scaled from 0.2 µm^2^ (deep red) to 12 µm^2^ (light green) across all experiments. For experiments presented in Fig. 6, the clones were detected and the circularity was measured using ImageJ. Ten *z*-slices from the centre of the stack were projected using maximum projection. Gaussian Blur filter (sigma=3.5) was applied and the mutant clones were identified based on loss of Ap staining using intensity thresholding (low intensity).

The mixing index and contact length (Fig. S2) were quantified using the dataset presented in Fig. 5. The number of clone border cells, number of wt contacting cells and clone perimeters were obtained from the CELL_CLONE Epitools module. Intensity measurements in Fig. S1 were performed using the ‘plot profile’ function of the Fiji image processing program.

### Statistical analysis

Data processing and statistical analysis were performed in R v3.5.0 and GraphPad. Conditions were compared using unpaired two-sampled Wilcoxon test (also known as Mann–Whitney test). Comparisons with a *P*-value ≥0.05 were marked as ‘ns’ (not significant); *P<0.05; **P<0.01; ***P<0.001. Note that for circularity measurements in Fig. 5K, as well as mixing index and contact length measurements in Fig. S2C,D, 12 out of 47 *sqhRNAi* data points were removed from the analysis. These corresponded to small GFP-positive groups of cells (consisting of one to ten cells), located close to bigger clones and, thus, they were presumed to be split from the bigger clones.

## Supplementary Material

10.1242/joces.259935_sup1 Supplementary information
