# Supplementary material for: Elimination of aberrantly specified cell clones is independent of interfacial Myosin II accumulation
Source: J Cell Sci. 2023 Jul 11;136(13):jcs259935. doi: 10.1242/jcs.259935 (PMC10357031; doi:10.1242/jcs.259935)
Supplement: Supplementary information [file joces-136-259935-s1.pdf]

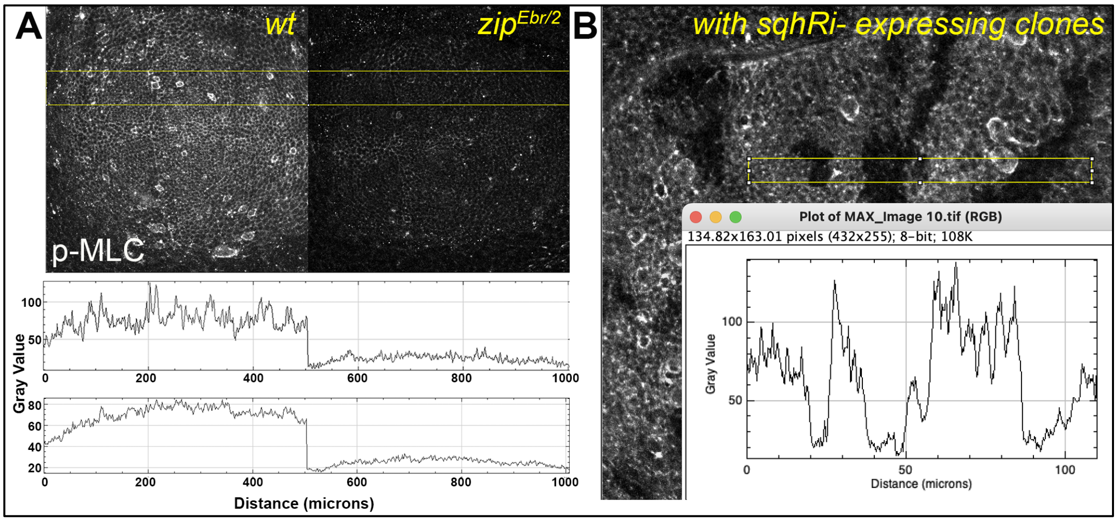

Fig. S1 (related to Figures 4 and 5). Quantification of residual p-MLC staining upon targeting the heavy and the light chains of Myosin II.

Intensity profiles obtained using the plot profile function of Fiji from the images shown. Averaged intensities (gray values on x-axis) of p-MLC staining plotted against distances in microns in y-axis. **A)** Above plot shows intensity quantification of p-MLC staining averaged over y-axis within the yellow box from wild-type (left) and *zipper* mutant (right) wing discs shown in Fig. 4A-B. Below plot shows average intensities from the entirety of the same images. Note that the residual p-MLC in *zip*$^{Ebr/2}$ wing disc does not exceed 25-30% of normal levels and approaches zero at the edges. **B)** Intensity profile corresponding to the region in yellow box from a disc expressing *sqhRi* in clones shown in Fig.5A-A'. Residual p-MLC in *sqhRi*-expressing cells do not exceed 20% of normal levels and approaches zero in the middle of the largest clone measured at 50-micron mark.

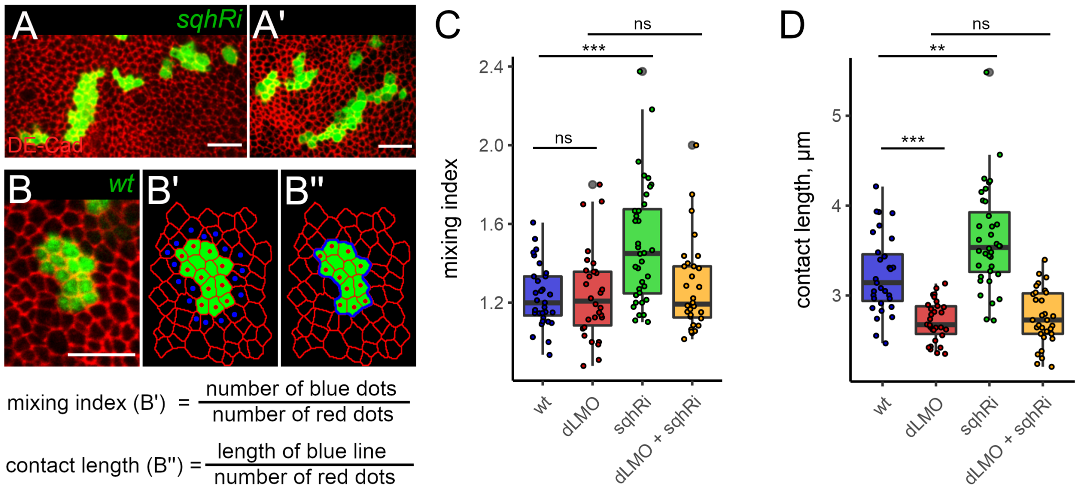

Fig. S2 (related to Figure 5). Cell clones expressing *sqh-RNAi* disperse and mix with their wild-type neighbours.

A-A') Examples of cell clones expressing *sqh-RNAi* (green) stained for DE-Cad (red). Such clones display a more dispersed morphology than wild-type clones. **B)** A GFP-marked (green) wild-type clone in a disc stained for DE-Cad (red). **B'-B'')** Schematics explain the logic used for driving the mixing index and contact length formulas shown below. Red dots show clonal cells touching the boundary, whereas blue dots mark wild-type cells touching the boundary. Clone outline (perimeter of the clone) is shown as a blue line in (B''). **C-D)** Quantification of the mixing index (C) and contact length (D) for clones of the indicated genotypes. Minimum 30 clones per genotype were analysed. Cells in *sqh-RNAi* clones contact a higher number of surrounding cells and these contacts are longer. *dLMO* expression on its own or along with *sqh-RNAi* reduces the contact length with the surrounding cells. Scale bars represent 10µm in all panels.

Journal of Cell Science • Supplementary information

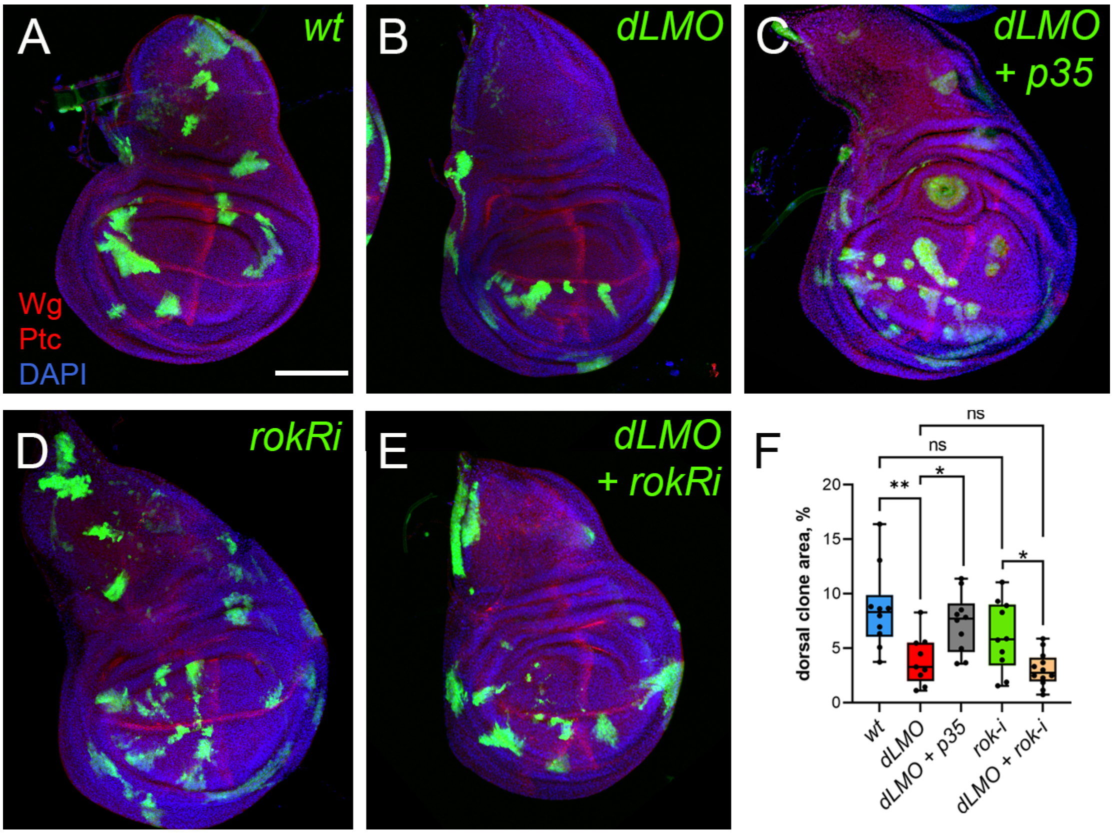

Fig. S3. Knocking down *rho kinase* within *dLMO*-expressing clones does not influence clone recovery

A-E) Wing discs containing control (A), *dLMO*-expressing (B), *dLMO* and *p35*-expressing (C), *rok-RNAi*-expressing (D), or *dLMO* and *rok-RNAi*-expressing clones (green) (E), stained for Wg (red), Ptc (red), and DAPI (blue). **F)** Quantification of dorsal clone area in indicated genotypes. Minimum 10 discs were analysed per genotype. All images are shown at the same magnification. The scale bar in (A) corresponds to 100µm. Dorsal is up, anterior is to the left in all panels.

Journal of Cell Science • Supplementary information

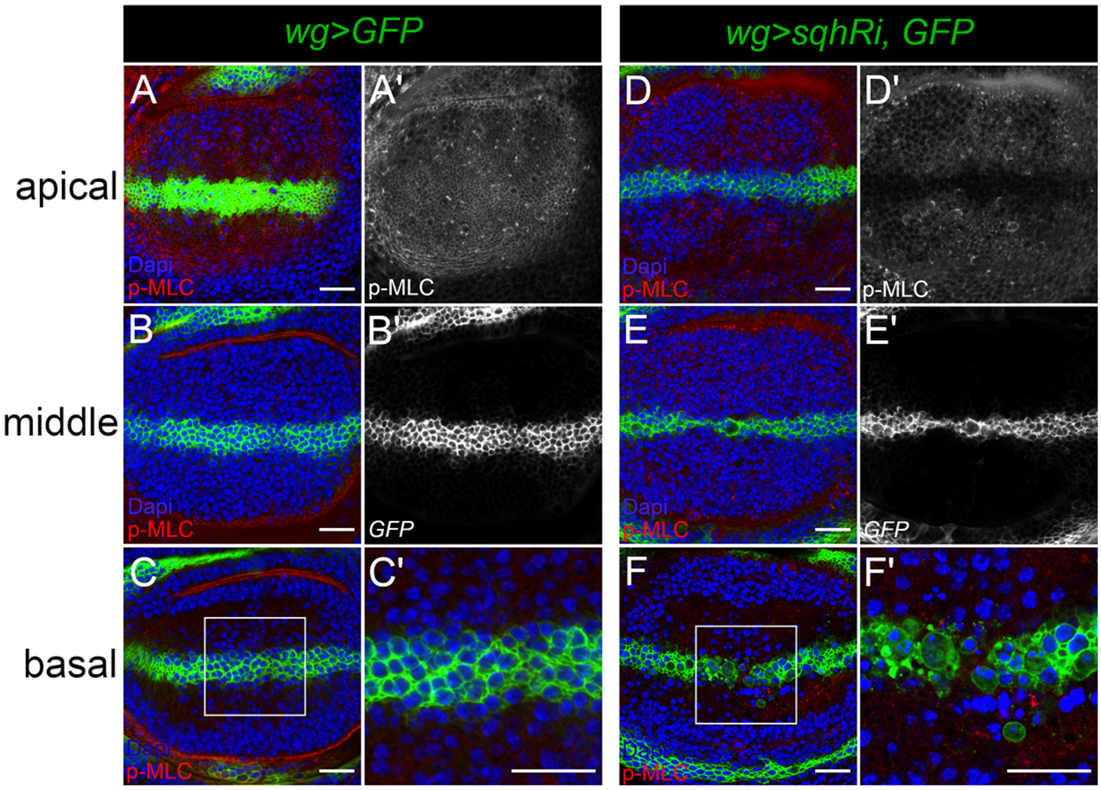

Fig. S4 (related to Figure 6). *sqh-RNAi* expression at the DV boundary undermines integrity of the boundary.

A-B) Pouch regions from representative discs expressing GFP (green) (left) or GFP and *sqh-RNAi* (right) under *wg::Gal4* control, stained for DAPI (blue) and p-MLC (red). Optical sections at three different levels are shown: apical, middle and basal. C' and F' shows zoomed versions of white boxes in C and F. A gap in the boundary and dying cells are visible at the basal side upon *sqh* depletion. Scale bars represent 20µm in all panels. Dorsal is up, anterior is to the left in all panels.

Journal of Cell Science • Supplementary information

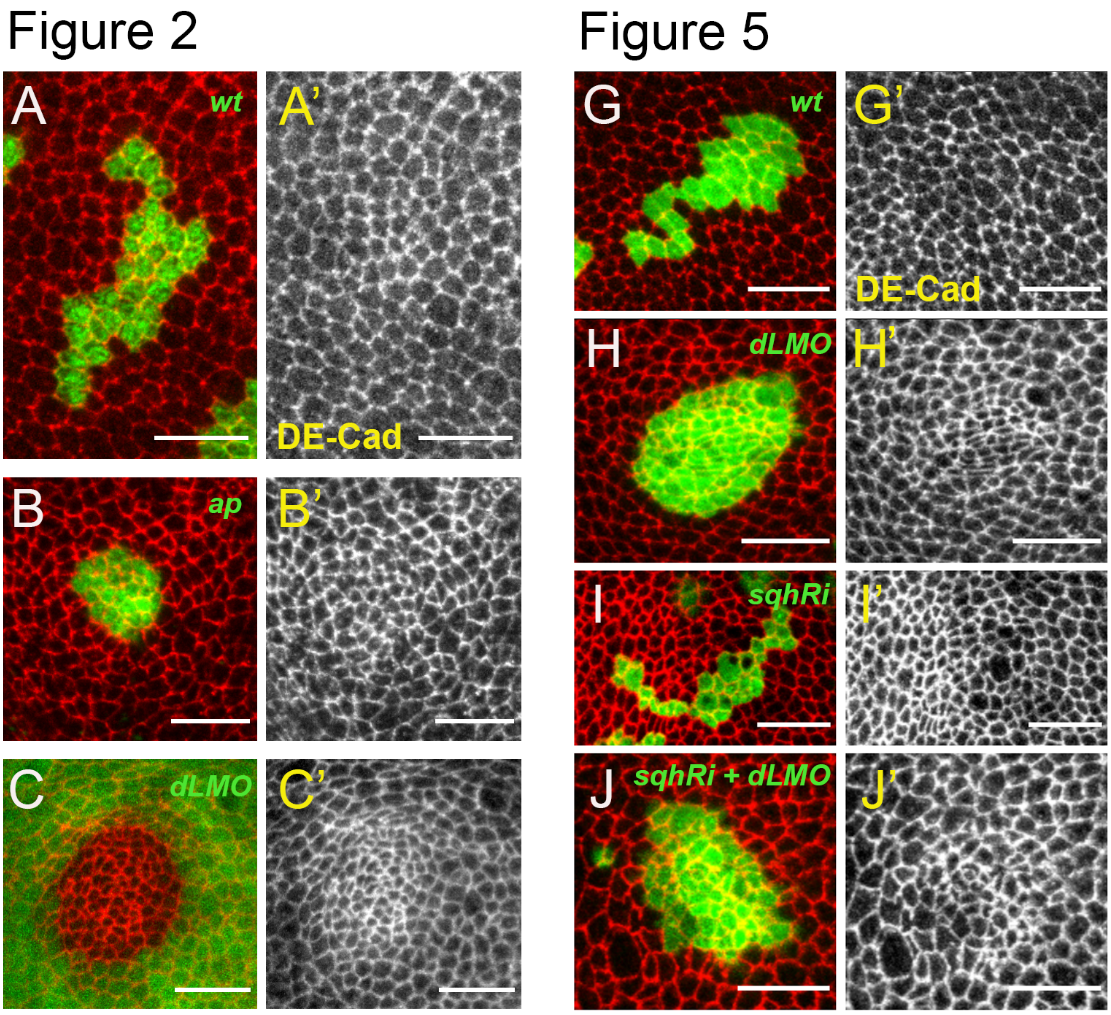

Fig. S5 (related to Figures 2 and 5). DE-Cad levels and localization are not affected in aberrantly specified cell clones. Corresponding panels from figures 2 (left) and 5 (right) are shown with individual DE-Cad channels in grayscale.

Table S1. Genotypes and experimental set-up for each figure panel

Figure	Genotype	Time AEL, h			Heat-shock duration, min
		**Egg collection**	**Heat-shock**	**Dissection**	
1B	*yw*	-	-	3rd instar	-
1C	*yw hsflp / yw;* FRT^{f00878} ap^{DG8} / FRT^{f00878} *tub-Gal80; tub-Gal4 UAS-GFP / +*	24	32-56	76-100	30
1D	*yw hsflp / w; UAS-Ap / +; act>CD2>Gal4 UAS-GFP / +*	4	42-46	86-90	12
1E	*yw hsflp / w; UAS-dLMO / +; act>CD2>Gal4 UAS-GFP / +*	5	55-60	100-105	50
1F	*yw hsflp / w; UAS-Ap / +; act>CD2>Gal4 UAS-GFP / +*	5	55-60	100-105	50
2A	*yw hsflp /yw;* FRT^{f00878}/ FRT^{f00878} *tub-Gal80; tub-Gal4 UAS-GFP/+*	8	50-58	92-100	30
2B	*yw hsflp /yw;* FRT^{f00878} ap^{DG8}/ FRT^{f00878} *tub-Gal80; tub-Gal4 UAS-GFP / +*	8	50-58	92-100	30
2C	*yw hsflp / w; UAS-dLMO / +; act>CD2>Gal4 UAS-GFP / +*	8	50-58	96-103	50
3A	*yw hsflp / yw; UAS-dLMO / +; act>CD2>Gal4 UAS-GFP / +*	8	61-69	112-120	13
3B	*yw hsflp / Y or sqh[AX3]; UAS-dLMO / sqh-GFP UAS-mCherry; act>CD2>Gal4 / +*	8	61-69	112-120	13
3C	*yw hsflp / yw; UAS-dLMO / +; act>CD2>Gal4 UAS-GFP / +*	8	61-69	112-120	50
3D	*yw hsflp / Y or sqh[AX3]; UAS-dLMO / sqh-GFP UAS-mCherry; act>CD2>Gal4 / +*	8	61-69	112-120	50
4A	*yw hsflp / yw; CyO Dfd-YFP / +; act>CD2>Gal4 UAS-GFP / +*	8	60-68	110-118	13
4B	*hsflp / w;* zip^{Ebr} / zip^{2} *; act>CD2>Gal4 UAS-GFP / +*	8	60-68	110-118	13
4C	*yw hsflp / w; + / CyO or IF; act>CD2>Gal4 UAS-GFP / MKRS or TM6B*	9	51-60	110-119	13
4D	*yw hsflp / w; UAS-dLMO / CyO or IF; act>CD2>Gal4 UAS-GFP / MKRS or TM6B*	9	51-60	110-119	13

Figure	Genotype	Time AEL, h			Heat-shock duration, min
		**Egg collection**	**Heat-shock**	**Dissection**	
4E	*yw hsflp / w; zip^{Ebr} / zip^{2}; act>CD2>Gal4 UAS-GFP / +*	9	51-60	110-119	13
4F	*yw hsflp / w; zip^{Ebr} / zip^{2}; act>CD2>Gal4 UAS-GFP / UAS-dLMO*	9	51-60	110-119	13
4H	*yw hsflp / yw; CyO Dfd-YFP / +; act>CD2>Gal4 UAS-GFP / +*	8	60-68	110-118	13
4I	*yw hsflp / yw; UAS-dLMO / +; act>CD2>Gal4 UAS-GFP / +*	8	60-68	110-118	13
4J	*yw hsflp / w; zip^{Ebr} / zip^{2} ; act>CD2>Gal4 UAS-GFP / +*	8	60-68	110-118	13
4K	*yw hsflp / w; zip^{Ebr} / zip^{2} ; act>CD2>Gal4 UAS-GFP / UAS-dLMO*	8	60-68	110-118	13
5A	*yw hsflp / w; CyO Dfd-YFP / +; act>CD2>Gal4 UAS-GFP / UAS-sqhRNAi*	8	47-55	90-98	13
5B	*yw hsflp / yw; CyO Dfd-YFP / +; act>CD2>Gal4 UAS-GFP / +*	8	51-59	112-120	13
5C	*yw hsflp / yw; UAS-dLMO / +; act>CD2>Gal4 UAS-GFP / +*	8	51-59	112-120	13
5D	*yw hsflp / w; CyO Dfd-YFP / +; act>CD2>Gal4 UAS-GFP / UAS-sqhRNAi*	8	51-59	112-120	13
5E	*yw hsflp / w; UAS-dLMO / +; act>CD2>Gal4 UAS-GFP / UAS-sqhRNAi*	8	51-59	112-120	13
5G	*yw hsflp / yw; CyO Dfd-YFP / +; act>CD2>Gal4 UAS-GFP / +*	8	58-66	108-116	13
5H	*yw hsflp / yw; UAS-dLMO / +; act>CD2>Gal4 UAS-GFP / +*	8	58-66	108-116	13
5I	*yw hsflp / w; CyO Dfd-YFP / +; act>CD2>Gal4 UAS-GFP / UAS-sqhRNAi*	8	58-66	108-116	13
5J	*yw hsflp / w; UAS-dLMO / +; act>CD2>Gal4 UAS-GFP / UAS-sqhRNAi*	8	58-66	108-116	13
6A	*(yw) hsflp / (y)w; wg::Gal4 FRT^{f00878} / FRT^{f00878} ap^{DG8} ; UAS-GFP / MKRS or TM6*	8	50-58	110-118 (Day5) (Day 6 in 6D-E 134-142)	30
6B-C	*(yw) hsflp / (y)w; wg::Gal4 FRT^{f00878} /*	8	50-58	110-118	30

Journal of Cell Science • Supplementary information

Figure	Genotype	Time AEL, h			Heat-shock duration, min
		**Egg collection**	**Heat-shock**	**Dissection**	
	FRT^{f00878} ap^{DG8} ; UAS-GFP / UAS-sqhRNAi			(Day5) (Day 6 in 6D-E 134-142)	
6F	*(yw) hsflp / (y)w; wg::Gal4 FRT^{f00878} / FRT^{f00878} ap^{DG8} ; UAS-GFP / MKRS or TM6*	8	61-69	110-118	30
6G	*(yw) hsflp / (y)w; wg::Gal4 FRT^{f00878} / FRT^{f00878} ap^{DG8} ; UAS-GFP / UAS-sqhRNAi*	8	61-69	110-118	30
7A	*yw hsflp /yw; FRT^{f00878} ap^{DG8}/ FRT^{f00878} tub-Gal80; tub-Gal4 UAS-GFP / +*	8	51-58	92-100	30
7B	*yw hsflp / w; zip^{Ebr} / zip^{2} ; act>CD2>Gal4 UAS-GFP / UAS-dLMO*	8	60-68	110-118	13
7C	*yw hsflp / w; UAS-dLMO / +; act>CD2>Gal4 UAS-GFP / UAS-sqhRNAi*	8	51-59	112-120	13
7D	*(yw) hsflp / (y)w; wg::Gal4 FRT^{f00878} / FRT^{f00878} ap^{DG8} ; UAS-GFP / UAS-sqhRNAi*	8	61-69	110-118	30
S2A-A′	*yw hsflp / w; CyO Dfd-YFP / +; act>CD2>Gal4 UAS-GFP / UAS-sqhRNAi*	8	58-66	108-116	13
S2B	*yw hsflp /yw; FRT^{f00878}/ FRT^{f00878} tub-Gal80; tub-Gal4 UAS-GFP/+*	8	50-58	92-100	30
S3-A	*yw hsflp / yw; CyO Dfd-YFP / +; act>CD2>Gal4 UAS-GFP / +*	8	50-58	112-120	13
S3-B	*yw hsflp / yw; UAS-dLMO / +; act>CD2>Gal4 UAS-GFP / +*	8	50-58	112-120	13
S3-C	*yw hsflp / yw; UAS-dLMO / +; act>CD2>Gal4 UAS-GFP / UAS-p35*	8	50-58	112-120	13
S3-D	*yw hsflp / yv ; CyO Dfd-YFP / +; act>CD2>Gal4 UAS-GFP / UAS-rokRNAi*	8	50-58	112-120	13
S3-E	*yw hsflp / yv; UAS-dLMO / +; act>CD2>Gal4 UAS-GFP / UAS-rokRNAi*	8	50-58	112-120	13
S4A-C	*(yw) hsflp / (yw); wg::Gal4 FRT^{f00878} / FRT^{f00878}; UAS-GFP / MKRS or TM6*	10	50-60	109-119	30
S4D-F	*(yw) hsflp / (yw); wg::Gal4 FRT^{f00878} / FRT^{f00878}; UAS-GFP / UAS-sqhRNAi*	10	50-60	109-119	30
