# Supplementary material for: Elimination of aberrantly specified cell clones is independent of interfacial Myosin II accumulation
Source: J Cell Sci. 2023 Jul 11;136(13):jcs259935. doi: 10.1242/jcs.259935 (PMC10357031; doi:10.1242/jcs.259935)
Supplement: Supplementary file 1 [file joces-136-259935_review_history.pdf]

Journal of Cell Science

Elimination of aberrantly specified cell clones is independent of interfacial Myosin II accumulation

Olga Klipa, Menna El Gammal and Fisun Hamaratoglu
DOI: 10.1242/jcs.259935

Editor: Andrew Ewald

Review timeline

Original submission:	18 February 2022
Editorial decision:	4 April 2022
First revision received:	20 March 2023
Editorial decision:	28 April 2023
Second revision received:	31 May 2023
Accepted:	4 June 2023

Original submission

First decision letter

MS ID#: JOCES/2022/259935

MS TITLE: Elimination of Aberrantly Specified Cell Clones Is Independent of Interfacial Myosin II Accumulation

AUTHORS: Olga Klipa, Mennat El Gammal, and Fisun Hamaratoglu
ARTICLE TYPE: Research Article

We have now reached a decision on the above manuscript.

To see the reviewers' reports and a copy of this decision letter, please go to: https://submit-jcs.biologists.org and click on the 'Manuscripts with Decisions' queue in the Author Area. (Corresponding author only has access to reviews.)

As you will see, the reviewers raise a number of substantial criticisms that prevent me from accepting the paper at this stage. They suggest, however, that a revised version might prove acceptable, if you can address their concerns. If you think that you can deal satisfactorily with the criticisms on revision, I would be pleased to see a revised manuscript. We would then return it to the reviewers. I note that my opinion is closer to that of Reviewer 1 than Reviewer 2. I anticipate (as specified by Reviewer 2) that many of the concerns raised by that reviewer about interpretation of myosin II depletion experiments can be addressed by careful rewriting of the text. I do think it is important to clarify the possibilities for how the system could work if the mechanism isn't myosin II independent (concern raised by Reviewer 1). In case it is helpful to you, I include below Reviewer 1's assessment (edited) of the comments by Reviewer 2:

Reviewer 1: "I do understand the concern of the referee 2 about the duty ratio of MyoII (and I think it would be important to mention this in the discussion), but I would like to stress the fact that in the field of epithelial morphogenesis, the vast majority of results published show clear correlation between junctional MyoII concentration and tension. Showing a context where MyoII levels does not match the expected distribution of tension is interesting by itself I believe and I don't think it lower down the interest of these results. Similary all the genetic context depleting MyoII and/or decreasing the levels of ROCK/Rho1 are associated with a decrease of cortical tension (as far as I

© 2023. Published by The Company of Biologists under the terms of the Creative Commons Attribution License (https://creativecommons.org/licenses/by/4.0/).

know, especially in Drosophila). So to the best of my knowledge, I never saw a clear example where 60-80% depletion of MyoII was compensated by the increase of MyoII duty ratio...(and I would be really surprised if this happens here)."

Please ensure that you clearly highlight all changes made in the revised manuscript. Please avoid using 'Tracked changes' in Word files as these are lost in PDF conversion.

I should be grateful if you would also provide a point-by-point response detailing how you have dealt with the points raised by the reviewers in the 'Response to Reviewers' box. Please attend to all of the reviewers' comments. If you do not agree with any of their criticisms or suggestions please explain clearly why this is so.

Reviewer 1

Advance summary and potential significance to field

In this manuscript, Klipa and colleagues explore the mechanism of elimination of miss-specified cells in the wing imaginal disc by perturbing genes normally associated with the sorting of the dorsal and ventral compartment (namely Apterous). Previous work demonstrated that clones wrongly expressing Apterous and/or lacking Apterous in the dorsal compartment were eliminated by apoptosis and extrusion (first described by Milan et al Dev Cell 2002, further characterised later on by the authors of this study Klipa and Hamaratoglu Plos genetics 2019). Clone elimination is also associated with cell sorting (rounding of clone boundary) cyst formation and exclusion of cell from the epithelial layer. Similar processes were described in many conditions associated with misspecification of cells (including Dpp and Wg), and more recently were suggested to be driven by the increase interface contractility and an increase of actomyosin at the interface which was supposed to be responsible for cell sorting and cyst formation (Bielmeier et al. Curr Biol 2016). While the link between sorting, interfacial tension and cell elimination was not formally demonstrated experimentally, it was suggested to be one putative explanation of cell extrusion and apoptosis, as the increase of line tension can generate a local increase of pressure (specially for low curvature as described by the Laplace law). Here, the authors test formally whether interfacial tension and myosin II are indeed responsible for sorting and the elimination of misspecified cells. Using a combination of elegant and demonstrative genetic perturbations, they surprisingly show that MyoII (and more specifically MyoII at the interface between the misspecified clone and the rest of thre tissue) is not required for sorting, nore clone elimination. The demonstration is based on a limited number of relativey Â“simpleÂ” experiments (here simple has nothing negative), yet highly demonstrative and convincing (specially when depleting MyoII at the interface between the two populations).

These results open essential question related to two central developmental biology problems : the formation of compartment boundary and cell sorting, and the mechanism of cell elimination by cell competition.

Lately, the field of epithelial morphogenesis has been dominated by a MyoII centric view of the regulation of cell deformation. This also includes cell sorting and the formation of compartment boundary which was assumed to be based on local increase of tension at the interface between the two populations through MyoII accumulation (Major and Irvine 2006, Monier et al. NCB 2010 Sanson lab, Landsberg et al. Curr Biol 2009, Dahmann group) or more recently the ratio of tension between the interface and the bulk of the two populations (Bosveld et al. 2016 Development Bellaiche lab). While this manuscript does not propose a concrete answer to how sorting is then achieved in the context of dorso-ventral mispecified cells, it stress the need for exploring alternative mechanism of sorting and cell elimination (either through alternative regulator of tension, or adhesive properties). I therefore believe that it is important to publish these results (even if they do not propose a clear mechanism) in order to encourage the exploration of alternative mechanisms by the community.

© 2023. Published by The Company of Biologists under the terms of the Creative Commons Attribution License (https://creativecommons.org/licenses/by/4.0/).

Comments for the author

I do have though some suggestions that could first clarify some part of the manuscript as well as the theoretical background of sorting. I would also have a couple suggestion for experiments that could complement the demonstration by clarifying the role of interfacial tension and excluding alternative mechanisms based on differential growth.

1. So far, the authors do not really discuss what could be the alternative mechanism for sorting and cell elimination. I believe it would be important to clearly delineate alternative mechanism in the discussion of the manuscript, which could act either through components affecting interfacial tension without visible modification of MyoII, or through alternative sorting mechanisms based on differential adhesion. Indeed, there are other regulators of cortical tension which may impact interfacial contractility without any visible change of MyoII (for instance Dachs was shown to play such role in the pupal notum, Bosveld et al. Science 2016 Bellaiche lab). Similarly, work in cell culture have shown that actin organization could impact junction tension without modification of local MyoII concentration (Chugh P et al, NCB 2017, Paluch lab, and a similar aspect was shown during cell competition, Levayer et al 2015 Moreno lab). Excluding the contribution of MyoII does not exclude a contribution of a local increase of interfacial tension, and I believe this should be clearly stated. Alternatively, adhesive components could also contribute to sorting and cell elimination (e.g: Iijima et al. Nat Comm 2020 and the role of Toll1), and it would be important to mention this alternative mechanism.

2. I believe it would help to introduce the concept of Laplace pressure (which relates interfacial tension curvature and pressure) when the authors describe the link between interfacial tension, pressure and cell elimination (which help to explain why smaller clones and the group in minority is the one eliminated) . Also it would be good to compare more thouroughly the results of this manuscript to the recent theoretical explorations showing that relative changes of tension between clone bulk, the rest of the tissue and the interface is sufficient to predict the level of sorting (Bosveld et al. Development 2016 Bellaiche lab). In that regard, this article and the concept behind may need to be introduce before the discussion to help the interpretation of the results.

3. In all the condition affecting sorting, could the author show the E-cad channel alone (notably in Figure 2 and Figure 5) ? Right now it is difficult to see if the local concentration of E-cad are affected in all these conditions and that could be an alternative mechanism affecting sorting.

4. Could the authors also show the distribution of actin in the context affecting MyoII (one may still see an enrichment of actin at the interface) ? This is especially relevant in the condition depleting sqh in the Wg domain on in the dLMO expressing clones.

5. A relative diminution of cell apical area in the clone and buckling could either be driven by external constraint and pressure, cell autonomous constriction or by a relative higher proliferation rate in the clone (which would also build up pressure, see for instance Pan et al. PNAS 2016, Irvine lab). In principle, one signature that could distinguish an apical area decrease driven by higher growth versus one induced by interfacial tension is the shape of the neighbouring cells: while in the first scenario they should be compressed radially and elongated tangentially along the clone interface (as shown in Legoff et al Development 2013 Lecuit lab, and Mao et al EMBO J 2013 Tapon lab), in the second scenario, they should be larger and elongated radially. Could the author inspect this in their segmented crops in the dLMO overexpressing clones ? Also, did they compare the rate of proliferation in the clone versus the rest of the tissue ? (it might have been done in their previous study, and if so it would be then interesting to refer to this result). Finally, it would be important to mention clearly that there is no obvious increase of MyoII in the bulk of the misspecified clone (which could have explained the reduced apical area).

6. As discussed above , relative changes of tension rather than absolute values might be more essential for sorting. In that regard, relative changes may even be stronger in conditions involving global downregulation of MyoII (e.g. : zipebr/zip2). An alternative way to flatten the differences could be through a global increase of tension/contractility. Could the authors characterize the elimination/sorting behaviour of misspecied clones upon a global increase of tension (e.g. : expressing an active form of ROCK, or sqhe20e21, or depleting the MyoII phosphatase Mbs in one compartment or throughout the wing pouch, combined with mitotic clone of Apterous mutant). Of

© 2023. Published by The Company of Biologists under the terms of the Creative Commons Attribution License (https://creativecommons.org/licenses/by/4.0/).

course this will not really address the physiological role of MyoII during sorting, but it may clearly test where relative changes of tension are still required for sorting/elimination.

Other minor point :
Page 5 top : I am not sure to understand what the authors means by “does not depend on […]the clone topological localisation” . It seems quite the contrary to me (the clone elimination purely relie on this localisation). This may need to be rephrased.

When measuring circularity, could the author also integrate the split cells in their measurement of perimeter/area ratio – taking the sum of perimeter and area of all the cell belonging to the same split clone (provided that clone are sparsed enough to unambiguously identify split clones) ? That could better reflect the degree of mixing/sorting (specially for the sqhRI condition).

Reviewer 2

Advance summary and potential significance to field

In this manuscript by Klipa et al., the authors explore how cells with different differentiation fates maintain the specified boundaries, including how they sort out or eliminate cells of the wrong fate within a given population. Using the Drosophila imaginal disc as a model, they explore whether nonmuscle myosin II (NMII), which conventionally is considered to maintain cell-cell junctions, is necessary to maintain these boundaries. By using wild type and NMII knockdowns they find that compartmentalization occurs even when NMII is knocked down suggesting that NMII is not required for maintaining these boundaries. In addition, apical constriction still occurs even when NMII is reduced.

The overall challenge in interpreting these data is that none of the manipulations are complete nulls. Therefore, the authors are forced to conclude that activity scales linearly with expression level and that the resulting phenotypes, or lack thereof, indicate that myosin II is or is not required. That could be true. However, this assumption neglects a couple of critical elements. First, myosin II is force-sensitive and increases duty ratio, up to 5-10 fold in response to mechanical forces. Thus, a 5-10-fold reduction in myosin II levels could be compensated by the 5-10-fold increased duty ratio for the remaining myosin II proteins. Second, and this is really a corollary of the first point force sharing is a fundamental feature of cytoskeletal systems. As a result, all the myosin II that is present at the junctions in the wild type scenario may not have to be doing as much due to load being distributed across a larger number of motors and other cytoskeletal proteins, such as actin crosslinkers. The consequence of these concerns is that the negative results from partial reduction in myosin levels are hard to interpret.

Comments for the author

Specific comments:
1. Page 6 and Fig. 3. The authors make assumptions that myosin II and actin levels are synonymous with “increased contractility”. In fact, that is not universally true. In many instances, the inverse is true due to force sharing and other properties myosin II brings to the actin network. I found this assumption to be concerning as so much is dependent on it in the subsequent sections.

2. Explanation of the two myosin II mutants is essential. How are the biochemical and biophysical properties of myosin II altered by these mutations?

Right now, the authors compare to the Major and Irvine paper, but that also does not explain what the mutations do to myosin II function. I presume that the mutations just lower the expression level and do not change other features, but this needs to be explicitly explained. Also, quantitative assessment on the expression levels is missing (this also comes up in points below).

3. Regarding Fig. 4A, B, p-MLC is considered a surrogate for total myosin II. Is that accurate for this system? What happens if you probe for myosin II heavy chain? Do both scale linearly with one another?

© 2023. Published by The Company of Biologists under the terms of the Creative Commons Attribution License (https://creativecommons.org/licenses/by/4.0/).

4. The observations in Fig. 4H-M really just mean that the level of myosin II activity still present in the zipper mutant combination is sufficient for the process. I cannot tell if this is zero myosin II activity or some value between zero and WT levels. Myosin IIs are also force-sensitive so one wonders if the function level that is present is picking up the slack in this case. Again, the response to point 2 above may help clarify.

5. For Fig. 5, it is good to see the RLC depletion, but the RLC is not 100% absent. What is the level of reduction, at least at the level of fluorescence differences? The statement is “very effective in depleting” is not especially quantitative. From the images, it is likely around a 70% reduction, which is probably not sufficient to interpret negative results.

6. Regarding statistical analysis, according to the Methods section, “two-sample t-tests” were used. This is incorrect for two reasons. First, many data sets incorporate more than two groups. Second, many distributions appear not to be normally (Gaussian) distributed. Therefore, two-or-more group analyses (e.g. parametric ANOVA or nonparametric Kruskal Wallis) should be used. For nonparametric, Wilcoxon should be used for follow up pair-wise comparisons.
Also, box-and-whiskers plots still obscure actual sample distributions. Please use dot plots so that all iterations may be observed.

7. Due to the statistical concern in point 6, it is possible that the impact of RLC knockdown (shqRi) is significantly different, even in a WT background.

8. The concept of “rounding” solely due to myosin II contractility is not a general idea. Other features like Laplace pressures are well known to drive cell rounding in a myosin II-independent manner. Therefore, one would not necessarily expect myosin II to be the only mechanisms for rounding of cells or groups of cells.

Minor comments:
1. Fig. 3: Arrows should be added to point out specific structures described. One can interpret a lot of things in these images in different ways and while I think I know what the authors are pointing out, please make it specifically clear.

Final Comments:
I think a lot of this can be addressed through re-writing and separating out the experimental observations from interpretations. Then, by incorporating the above considerations, they can provide a more rigorous interpretation in the Discussion. Quantification of the knockdown levels is also essential. My suspicion is that the knockdown scenarios are only ~70-80% effective, which could mean that there is ~20-30% of functional myosin II still in the cells. The residual level might have ample dynamic range to close the gap given the load-dependency of myosin II duty ratio and other force-sharing machinery that is undoubtedly present here.

<u>Reviewer 3</u>

Advance summary and potential significance to field

In this manuscript, Hamaratoglu and colleagues utilize the wing primordium of Drosophila to analyze the role of non-muscle Myosin II, known to regulate contractile forces in a cell, in the sorting and elimination of mis-specified cells. The wing primordium is subdivided into a dorsal and a ventral compartment, which will give rise to the two surfaces of the adult wing, and the boundary between these two populations is well known to be maintained by the activity of Myosin II. Clones of cells of dorsal or ventral identify induced in opposite compartments are round, their boundaries are smooth and clones are lost by apoptosis. Here, authors present convincing experimental evidence that Myosin II depletion does not interfere with the loss of these clones and does not rescue the shape and smoothness of these clones. They have carried out these experiments with the use of different tools (UAS-transgenes, mutant alleles) and have manipulated MyoII activity in the different cell populations. These data clearly contribute to our understanding of the molecular mechanisms underlying the removal of misspecified cells in development and open new avenues towards the identification of the real actors.

© 2023. Published by The Company of Biologists under the terms of the Creative Commons Attribution License (https://creativecommons.org/licenses/by/4.0/).

Comments for the author

Overall, the ms is beautifully written, the figures are clear, self-explanatory and nicely presented and organized and the discussion summarizes and contextualized in a smart way the results obtained in this study. Thus, my suggestion is a straightforward acceptance of the paper without any further delay.

I congratulate authors for this nice piece.

Minor comments:
(1) In the second paragraph of the Introduction, I would suggest to include references in all sentences to acknowledge the work of previous labs.
(2) In the fourth paragraph of the Introduction, the names of transcription factors should not be in italics and the first letter a capital one).
(3) Authors might want include the reference of Becam et al 2011 where the role of Myosin II in DV boundary formation is further analyzed.

First revision

Author response to reviewers' comments

We thank all reviewers for their constructive criticism and their suggestions. We will first highlight the main changes we implemented and then address each remark one by one.

Reviewer 3 was happy with our manuscript as is.

Reviewer 2 was adamant that remaining Myosin could be sufficient to generate enough force in our hypomorphic heavy chain mutant discs and upon RNAi driven knock-down of the light chain. We now provide quantifications of p-MLC in those conditions (Figure S1, shown below). The reviewer stated that “I think a lot of this can be addressed through re-writing and separating out the experimental observations from interpretations.” We believe that, we have now implemented this suggestion. All changes in the text and new text are highlighted in blue.

Moreover, we now have updated Figures 2, 4, 5, 6, S2 (former S1) and S3 (former S4) where we show all individual data points on top of box plots. We also re-did all our statistical analysis with a non- parametric test following Reviewer 2’s advice. New analysis caused only mild changes and we highlight those below.

Reviewer 1 was supportive of our main conclusions, and wanted us to better discuss alternative mechanisms in the absence of Myo-II driven interfacial tension, highlighting the examples in the recent literature. Reviewer 1 also wanted us to show DE-Cad channels alone to address a potential contribution of DE-Cad. We provide this in our new Figure S5 shown below.

We have two new supplemental figures S1 and S5:

© 2023. Published by The Company of Biologists under the terms of the Creative Commons Attribution License (https://creativecommons.org/licenses/by/4.0/).

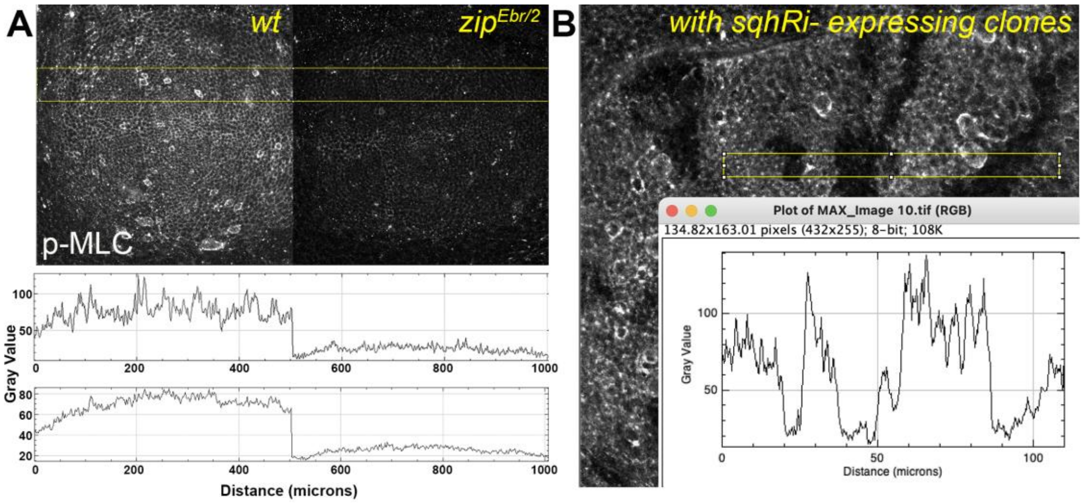

Figure S1 (related to Figures 4 and 5): Quantification of residual p-MLC staining upon targeting the heavy and the light chains of Myosin II.
Intensity profiles obtained using the plot profile function of Fiji from the images shown. Averaged intensities (gray values on x-axis) of p-MLC staining plotted against distances in microns in y-axis. **A)** Above plot shows intensity quantification of p-MLC staining averaged over y-axis within the yellow box from wild-type (left) and *zipper* mutant (right) wing discs shown in Fig. 4A-B. Below plot shows average intensities from the entirety of the same images. Note that the residual p-MLC in $zip^{Ebr/2}$ wing disc does not exceed 25-30% of normal levels and approaches zero at the edges. **B)** Intensity profile corresponding to the region in yellow box from a disc expressing *sqhRi* in clones shown in Fig.5A-A'. Residual p-MLC in *sqhRi*-expressing cells do not exceed 20% of normal levels and approaches zero in the middle of the largest clone measured at 50-micron mark.

© 2023. Published by The Company of Biologists under the terms of the Creative Commons Attribution License (https://creativecommons.org/licenses/by/4.0/).

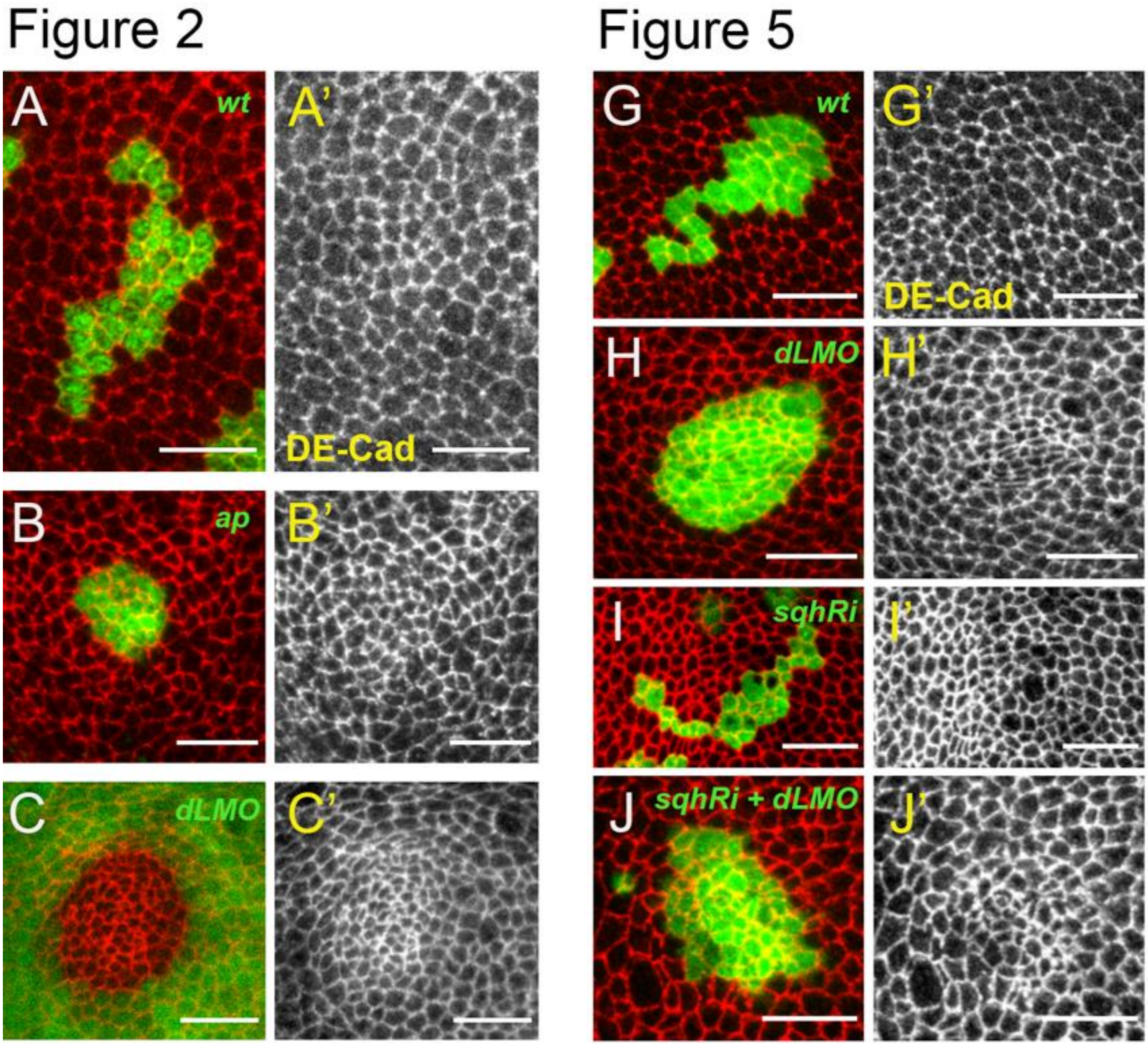

Figure S5 (related to Figures 2 and 5): DE-Cad levels and localization are not affected in aberrantly specified cell clones. Corresponding panels from figures 2 (left) and 5 (right) are shown with individual DE-Cad channels in grayscale.

We have added the following section to our Discussion (page 13) following Reviewer 1's advice: "What could be driving the increased tension at the clone boundary and sorting if Myosin II accumulation is not the sole contributor? One possibility is the differential adhesion, which was the first mechanism of cell sorting proposed and it has been backed with extensive modelling work as well as *in vitro* experiments (STEINBERG, 1963, Foty and Steinberg, 2005, Glazier and Graner, 1993). Recent work showed that differential expression of the transmembrane receptor Toll-1 in posterior cells contributes to a straight A/P boundary in the pupal abdominal epithelium in *Drosophila* (Iijima et al., 2020). We observed that some cell clones had increased concentration of DE-Cad, which could contribute to sorting, but this was not a general phenomenon and most of our aberrantly specified cell clones with smooth boundaries did not show differential DE-Cad expression (Fig. S5). Thus, adhesion molecules other than DE-cadherin could play a role in the process. Secondly, there are examples of actin cytoskeleton being reorganized without obvious changes in Myo-II activity in *Drosophila* (Levayer et al., 2015), and cultured cells (Chugh et al., 2017). Hence, we wondered whether actin filaments still accumulated around aberrantly specified cells with reduced Myo-II activity. Like the case with DE-Cad levels, actin accumulation was visible around a few clones, but the effect was not consistent. Therefore, we conclude that Myo-II independent actin accumulation is unlikely to contribute to the elimination of dLMO-expressing cell clones. Thirdly, differential localization of atypical myosin Dachs (D) could also generate tension. In the larval wing disc and pupal notum, D is polarized to one side of the cell

© 2023. Published by The Company of Biologists under the terms of the Creative Commons Attribution License (https://creativecommons.org/licenses/by/4.0/).

(Ambegaonkar et al., 2012, Brittle et al., 2012, Bosveld et al., 2012). Bosveld et al. found that junctions with D enrichment is under two-fold higher tension compared to junctions devoid of D in pupal notum (Bosveld et al., 2012). Notably, Dachs binds to actin filaments, but its motor domain cannot bind to ATP and hydrolyse it (Cao et al., 2014). Hence, how it generates force is unclear. Nevertheless, loss-of-function cell clones of the tumour suppressor gene *fat* rounds up in a Dachs dependent manner in the larval wing disc and the pupal notum (Mao et al., 2013, Mao et al., 2006, Bosveld et al., 2016, Bosveld et al., 2012). It will be important to investigate whether the rounding and clearing of aberrantly specified cell clones depend on Dachs activity."

Point by point response to Reviewers' comments:

Reviewer 1 Advance Summary and Potential Significance to Field:
In this manuscript, Klipa and colleagues explore the mechanism of elimination of miss- specified cells in the wing imaginal disc by perturbing genes normally associated with the sorting of the dorsal and ventral compartment (namely Apterous). Previous work demonstrated that clones wrongly expressing Apterous and/or lacking Apterous in the dorsal compartment were eliminated by apoptosis and extrusion (first described by Milan et al Dev Cell 2002, further characterised later on by the authors of this study Klipa and Hamaratoglu Plos genetics 2019). Clone elimination is also associated with cell sorting (rounding of clone boundary) cyst formation and exclusion of cell from the epithelial layer. Similar processes were described in many conditions associated with misspecification of cells (including Dpp and Wg), and more recently were suggested to be driven by the increase interface contractility and an increase of actomyosin at the interface which was supposed to be responsible for cell sorting and cyst formation (Bielmeier et al. Curr Biol 2016). While the link between sorting, interfacial tension and cell elimination was not formally demonstrated experimentally, it was suggested to be one putative explanation of cell extrusion and apoptosis, as the increase of line tension can generate a local increase of pressure (specially for low curvature, as described by the Laplace law). Here, the authors test formally whether interfacial tension and myosin II are indeed responsible for sorting and the elimination of misspecified cells. Using a combination of elegant and demonstrative genetic perturbations, they surprisingly show that MyoII (and more specifically MyoII at the interface between the misspecified clone and the rest of thre tissue) is not required for sorting, nore clone elimination. The demonstration is based on a limited number of relativey "simple" experiments (here simple has nothing negative), yet highly demonstrative and convincing (specially when depleting MyoII at the interface between the two populations). These results open essential question related to two central developmental biology problems : the formation of compartment boundary and cell sorting, and the mechanism of cell elimination by cell competition.Lately, the field of epithelial morphogenesis has been dominated by a MyoII centric view of the regulation of cell deformation. This also includes cell sorting and the formation of compartment boundary which was assumed to be based on local increase of tension at the interface between the two populations through MyoII accumulation (Major and Irvine 2006, Monier et al. NCB 2010 Sanson lab, Landsberg et al. Curr Biol 2009, Dahmann group) or more recently the ratio of tension between the interface and the bulk of the two populations (Bosveld et al. 2016 Development Bellaiche lab). While this manuscript does not propose a concrete answer to how sorting is then achieved in the context of dorso-ventral mispecified cells, it stress the need for exploring alternative mechanism of sorting and cell elimination (either through alternative regulator of tension, or adhesive properties). I therefore believe that it is important to publish these results (even if they do not propose a clear mechanism) in order to encourage the exploration of alternative mechanisms by the community.

Reviewer 1 Comments for the Author:
I do have though some suggestions that could first clarify some part of the manuscript as well as the theoretical background of sorting. I would also have a couple suggestion for experiments that could complement the demonstration by clarifying the role of interfacial tension and excluding alternative mechanisms based on differential growth.

1. So far, the authors do not really discuss what could be the alternative mechanism for sorting and cell elimination. I believe it would be important to clearly delineate alternative mechanism in the discussion of the manuscript, which could act either through components affecting interfacial tension without visible modification of MyoII, or through alternative sorting

© 2023. Published by The Company of Biologists under the terms of the Creative Commons Attribution License (https://creativecommons.org/licenses/by/4.0/).

mechanisms based on differential adhesion. Indeed, there are other regulators of cortical tension which may impact interfacial contractility without any visible change of MyoII (for instance, Dachs was shown to play such role in the pupal notum, Bosveld et al. Science 2016 Bellaiche lab). Similarly, work in cell culture have shown that actin organization could impact junction tension without modification of local MyoII concentration (Chugh P et al, NCB 2017, Paluch lab, and a similar aspect was shown during cell competition, Levayer et al 2015 Moreno lab). Excluding the contribution of MyoII does not exclude a contribution of a local increase of interfacial tension, and I believe this should be clearly stated. Alternatively, adhesive components could also contribute to sorting and cell elimination (e.g: Iijima et al. Nat Comm 2020 and the role of Toll1), and it would be important to mention this alternative mechanism.

We fully agree with the reviewer and thank them for pointing out this short-coming. Indeed, we show that the tension at the clone boundaries have not been released in our experimental conditions, evidenced with high circularity. We have included the above quoted section in our Discussion (page13) to cover the alternative mechanisms mentioned.

2. I believe it would help to introduce the concept of Laplace pressure (which relates interfacial tension, curvature and pressure) when the authors describe the link between interfacial tension, pressure and cell elimination (which help to explain why smaller clones and the group in minority is the one eliminated) . Also it would be good to compare more thouroughly the results of this manuscript to the recent theoretical explorations showing that relative changes of tension between clone bulk, the rest of the tissue and the interface is sufficient to predict the level of sorting (Bosveld et al. Development 2016 Bellaiche lab). In that regard, this article and the concept behind may need to be introduce before the discussion to help the interpretation of the results.

Thank you! We now introduce the Bosveld paper (formerly Ref 51) in the Introduction (page 5), we then further talk about it in the Discussion.

"Importantly, when considering segregation of clonal populations from their neighbours, it is also important to consider potential cell junction tension changes in the bulk of the clone, which were shown to contribute to sorting along with changes at the clonal boundary (Bosveld et al., 2016)."

We now introduce Laplace law in page 6 while describing the model we are testing. The new text is highlighted in blue.

"This suggests that among two differently fated cell populations that interact with each other, the underrepresented one is designated for elimination regardless of its identity. When one cell population is underrepresented, the boundary it forms with its surrounding neighbors is circular. In contrast, the compartment boundary, which is formed between relatively big and similarly sized cell populations, is linear. The former is associated with apoptosis and elimination whereas no cell death is induced in the latter. Thus, it is possible that the presence of relatively small groups of cells that disrupt global pattern (misspecified cells) could be detected by a circular interface. The underlying mechanism as to how interface shape determines the output could be attributed to the Laplace pressure and interface contractility. The Laplace pressure, which refers to the pressure difference between the inside and the outside of a curved boundary, is inversely proportional to the radius of the boundary. Therefore, a smaller clone is under higher Laplace pressure exerted by its boundary. According to this model, increased contractility around relatively small cell clusters causes irresistible mechanical stress (compression and apical constriction) in this encircled groups of cells, eventually leading to induction of apoptosis, and subsequent elimination (Bielmeier et al., 2016)."

3. In all the condition affecting sorting, could the author show the E-cad channel alone (notably in Figure 2 and Figure 5)? Right now it is difficult to see if the local concentration of E-cad are affected in all these conditions and that could be an alternative mechanism affecting sorting.

We have provided DE-cad only channels from the Figure 2 and 5 in Supp Fig 5. Indeed, we see accumulation of E-cad in some instances, but the effect is not consistent. For example, in 2C' there is convincing accumulation of E-cad in the wt island cells. However, the effect in Figure 5H' is more modest and is not restrained to the dLMO-expressing cells, surrounding cells above the clone also have high E-cad levels. Thus, we cannot exclude a contribution from enhanced adhesion

© 2023. Published by The Company of Biologists under the terms of the Creative Commons Attribution License (https://creativecommons.org/licenses/by/4.0/).

within the clone, but evident E-cad accumulation was observed in only a minority of the dLMO-expressing cell clones. We included this in the Discussion (page 13).

4.Could the authors also show the distribution of actin in the context affecting MyoII (one may still see an enrichment of actin at the interface)? This is especially relevant in the condition depleting sqh in the Wg domain on in the dLMO expressing clones.

We looked at many wing discs (20-30) that express sqh-RNAi or rok-RNAi in UAS-dLMO clones and show below the only example that shows convincing actin enrichment in or around the clones. Thus, actin may still accumulate around aberrantly specified clones after targeting Myo-II, but only occasionally and not consistently.

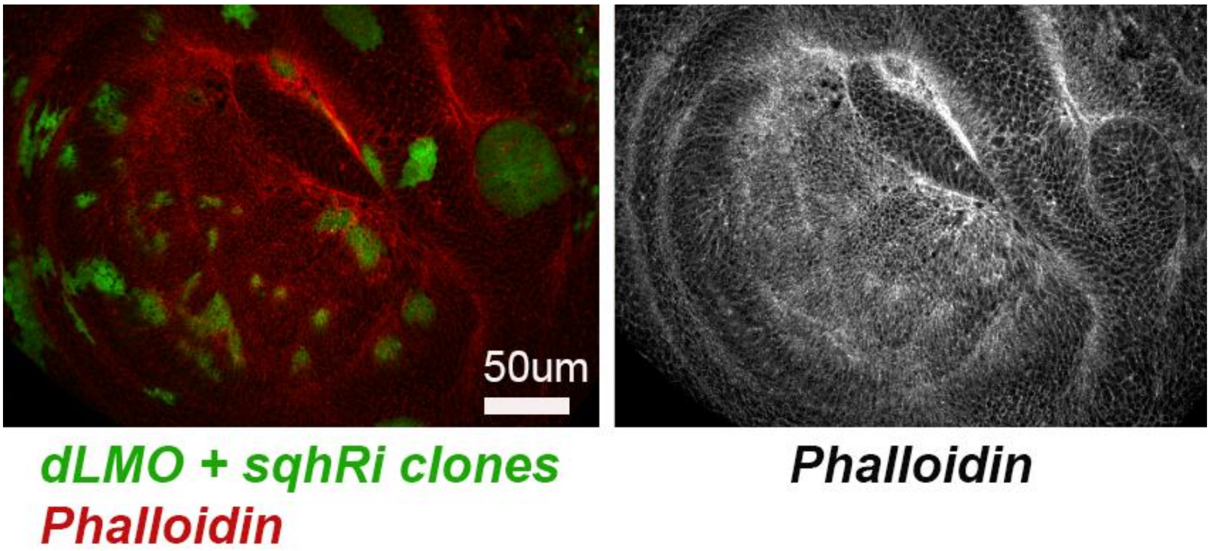

5. A relative diminution of cell apical area in the clone and buckling could either be driven by external constraint and pressure, cell autonomous constriction or by a relative higher proliferation rate in the clone (which would also build up pressure, see for instance Pan et al. PNAS 2016, Irvine lab). In principle, one signature that could distinguish an apical area decrease driven by higher growth versus one induced by interfacial tension is the shape of the neighbouring cells: while in the first scenario they should be compressed radially and elongated tangentially along the clone interface (as shown in Legoff et al Development 2013 Lecuit lab, and Mao et al EMBO J 2013 Tapon lab), in the second scenario, they should be larger and elongated radially. Could the author inspect this in their segmented crops in the dLMO overexpressing clones ? Also, did they compare the rate of proliferation in the clone versus the rest of the tissue ? (it might have been done in their previous study, and if so it would be then interesting to refer to this result).

Indeed, we analysed the proliferation pattern in the presence of aberrantly specified cell clones in our former work. The relevant figure is reproduced below. We do not see an increase in cell proliferation that is cell-autonomous, thus we do not think that differential proliferation contributes to the increased tension around mis-specified clones. In the dorsal hinge, both the clonal cells and the surrounding epithelia undergo additional divisions. Thus, the extra pressure this excessive proliferation generates would not correspond to the clone periphery.

In the dorsal pouch, we did not detect any changes in proliferation.

© 2023. Published by The Company of Biologists under the terms of the Creative Commons Attribution License (https://creativecommons.org/licenses/by/4.0/).

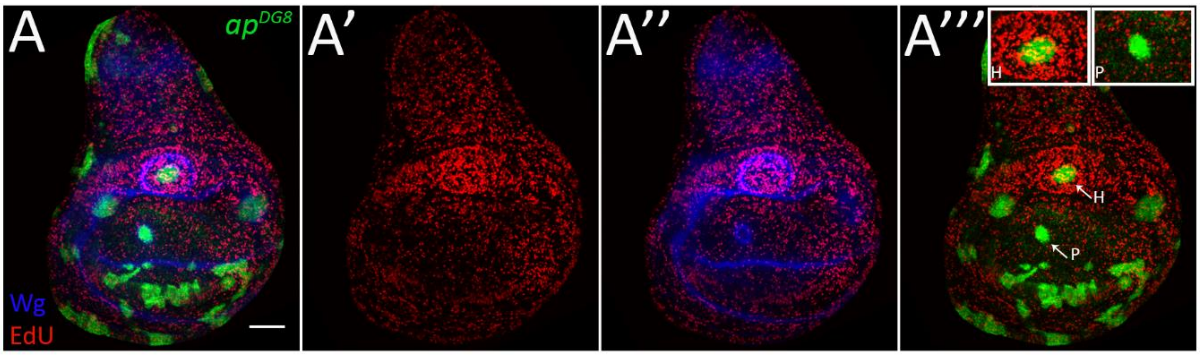

Figure S6 [from Klipa, O. and Hamaratoglu, F. (2019), PLOS Genetics 15, e1008573 (doi: 10.1371/journal.pgen.1008573), where it was published under the terms of a CC BY 4.0 licence]. *ap* mutant clones increase cell proliferation in the dorsal hinge but not in the dorsal pouch.

EdU cell proliferation assay of the third instar wing disc containing ap^{DG8} clones. (A) – merged image (ap^{DG8} clones, EdU and Wg staining); (A') – EdU channel alone; (A'') – EdU and Wg channels; (A''') – ap^{DG8} clones and EdU staining. The insets show enlarge images of single clones from dorsal pouch (P) and dorsal hinge (H). Scale bar represents 50μm.

Finally, it would be important to mention clearly that there is no obvious increase of MyoII in the bulk of the misspecified clone (which could have explained the reduced apical area).

We occasionally do see increased MyoII in the bulk of the aberrantly specified clones. We modified the following section to include that observation (new text is shown in blue):

Page 7: "Interestingly, in some cases elevated levels of Myosin were observed not only along the interface junctions but also at the junctions inside the misspecified clones, which may contribute to apical constrictions (Fig. 7A-A')."

6.As discussed above , relative changes of tension rather than absolute values might be more essential for sorting. In that regard, relative changes may even be stronger in conditions involving global downregulation of MyoII (e.g. : zipebr/zip2). An alternative way to flatten the differences could be through a global increase of tension/contractility. Could the authors characterize the elimination/sorting behaviour of misspecied clones upon a global increase of tension (e.g. : expressing an active form of ROCK, or sqhe20e21, or depleting the MyoII phosphatase Mbs in one compartment or throughout the wing pouch, combined with mitotic clone of Apterous mutant). Of course this will not really address the physiological role of MyoII during sorting, but it may clearly test where relative changes of tension are still required for sorting/elimination.

The reviewer raises an interesting possibility. As the reviewer points out we focus on the physiological role of Myo II in aberrantly specified cell elimination and thus we feel that the suggestion is outside the scope of this manuscript.

Other minor point :

Page 5 top : I am not sure to understand what the authors means by "does not depend on […]the clone topological localisation" . It seems quite the contrary to me (the clone elimination purely relie on this localisation). This may need to be rephrased.

Thanks for spotting this! We agree that cell death is observed predominantly in the pouch. We have rephrased. The new sentence reads:
page 5: "Both *ap* mutant cells in the dorsal compartment and Ap-expressing cells in the ventral compartment show strong induction of apoptosis (Fig. 1C-D', arrows). Thus, the apoptosis observed purely relies on cell-cell interactions, and does not depend on cell identity."

When measuring circularity, could the author also integrate the split cells in their measurement of

© 2023. Published by The Company of Biologists under the terms of the Creative Commons Attribution License (https://creativecommons.org/licenses/by/4.0/).

perimeter/area ratio – taking the sum of perimeter and area of all the cell belonging to the same split clone (provided that clone are sparsed enough to unambiguously identify split clones)? That could better reflect the degree of mixing/sorting (specially for the sqhRI condition).

Unfortunately, it's not possible to assign split clones to larger clones reliably. In many cases, the split clones are close enough to be part of more than one large clone. To reiterate, we used circularity as a representation of interfacial tension between two cell groups. In this context the individual cells that are not a part of the group could be considered as different groups (and therefore with different interfacial tensions), that is why we could not simply sum up the area / perimeter even if we could assign split clones reliably. However, we did highlight the differences in mixing/sorting by the introducing the mixing index (FigS2).

Reviewer 2 Advance Summary and Potential Significance to Field:
In this manuscript by Klipa et al., the authors explore how cells with different differentiation fates maintain the specified boundaries, including how they sort out or eliminate cells of the wrong fate within a given population. Using the Drosophila imaginal disc as a model, they explore whether nonmuscle myosin II (NMII), which conventionally is considered to maintain cell-cell junctions, is necessary to maintain these boundaries. By using wild type and NMII knockdowns, they find that compartmentalization occurs even when NMII is knocked down, suggesting that NMII is not required for maintaining these boundaries. In addition, apical constriction still occurs even when NMII is reduced. The overall challenge in interpreting these data is that none of the manipulations are complete nulls. Therefore, the authors are forced to conclude that activity scales linearly with expression level and that the resulting phenotypes, or lack thereof, indicate that myosin II is or is not required. That could be true. However, this assumption neglects a couple of critical elements. First, myosin II is force-sensitive and increases duty ratio, up to 5-10 fold in response to mechanical forces. Thus, a 5-10-fold reduction in myosin II levels could be compensated by the 5-10-fold increased duty ratio for the remaining myosin II proteins. Second, and this is really a corollary of the first point, force sharing is a fundamental feature of cytoskeletal systems. As a result, all the myosin II that is present at the junctions in the wild type scenario may not have to be doing as much due to load being distributed across a larger number of motors and other cytoskeletal proteins, such as actin crosslinkers. The consequence of these concerns is that the negative results from partial reduction in myosin levels are hard to interpret.

The reviewer is perfectly correct as we acknowledged clearly in the Discussion. We have been very careful not to say that the process was Myosin-independent. This is also reflected on our title "Elimination of Aberrantly Specified Cell Clones Is Independent of Interfacial Myosin II Accumulation". We are fully aware that we cannot work with null conditions as Myosin is essential, but we do revert Myosin accumulation at the boundary, and conclude "rounding and elimination of misspecified clones do not rely on Myosin accumulation along the boundary between differently fated cells" (page13). To ensure that we take the reviewer's comment into consideration, we have now replaced "Myosin II" with "Myosin II accumulation" in the text. Such changes are highlighted in blue.

For example:

Abstract: "Here we test whether interfacial tension driven by accumulation of Myosin is responsible for the elimination of aberrantly specified cells that would otherwise compromise compartment organization."

Page 14: "Thus, we have ruled out a decisive role for Myosin II accumulation driven tension in elimination of mispositioned cell clusters."

Reviewer 2 Comments for the Author:
Specific comments:
1.Page 6 and Fig. 3. The authors make assumptions that myosin II and actin levels are synonymous with "increased contractility". In fact, that is not universally true. In many instances, the inverse is true due to force sharing and other properties myosin II brings to the actin network. I found this assumption to be concerning as so much is dependent on it in the subsequent sections.

© 2023. Published by The Company of Biologists under the terms of the Creative Commons Attribution License (https://creativecommons.org/licenses/by/4.0/).

In the literature, increased contractility is closely associated with accumulation of actomyosin. Thus, the assumption is not ours, we are describing the predominant model and testing it genetically. Our conclusion is that interfering with Myosin accumulation does not relieve the tension at the boundary.

2. Explanation of the two myosin II mutants is essential. How are the biochemical and biophysical properties of myosin II altered by these mutations? Right now, the authors compare to the Major and Irvine paper, but that also does not explain what the mutations do to myosin II function. I presume that the mutations just lower the expression level and do not change other features, but this needs to be explicitly explained. Also, quantitative assessment on the expression levels is missing (this also comes up in points below).

We thank the reviewer for this suggestion. We quantified the levels of P-MLC in normal vs zip mutant discs using the plot profile function of Fiji (Figure S1A). We have included this information in the text (page 7):

"Non-muscle Myosin II is a vital molecule and its function is highly important for development. That is why animals bearing a strong Myosin mutation are not viable. However, some combinations of hypomorphic alleles of Myosin heavy chain *zipper (zip)* are less harmful and flies can survive until later stages. It was shown that zip^{Ebr}/zip^{2} is the strongest combination that allows recovery of third instar larvae (Major and Irvine, 2006). We assessed Myosin levels in the zip^{Ebr}/zip^{2} wing discs using antibodies against phosphorylated form of Myosin light chain (p-MLC), which is an indicator of assembled active form of the Myosin complex. In zip^{Ebr}/zip^{2} wing discs, Myosin levels were greatly reduced; the residual p-MLC did not exceed 25-30% when compared to wt discs (Fig. 4A, 4B, and S1A)."

We cite the papers that describe these alleles in the Materials and Methods. One of these papers (Halsell et al., 2000) analyzed Zipper protein levels in homozygous mutant embryos. They show that zip^{2} allele (premature stop codon at residue 750) is likely to be a protein null as there is no signal corresponding to Zipper in the western blot, while zip^{Ebr} (a missense mutation in exon 9, mutating a conserved arginine near the ATP binding pocket) allele leads to a about 50% reduction in overall Zipper protein levels.

3. Regarding Fig. 4A, B, p-MLC is considered a surrogate for total myosin II. Is that accurate for this system? What happens if you probe for myosin II heavy chain? Do both scale linearly with one another?

We did not use P-MLC antibodies as a surrogate for total Myosin, but rather as a readout for Myosin activity.

We obtained Zipper antibodies (described in Chougule et al., 2016) from the Thomas Lab (Texas Tech University Health Sciences Center), and probed our hypomorphic mutant discs, and were able to detect Zipper signal in mutant discs as expected. However, the staining was too noisy for quantification.

4. The observations in Fig. 4H-M really just mean that the level of myosin II activity still present in the zipper mutant combination is sufficient for the process. I cannot tell if this is zero myosin II activity or some value between zero and WT levels. Myosin IIs are also force-sensitive so one wonders if the function level that is present is picking up the slack in this case. Again, the response to point 2 above may help clarify.

We fully agree with the reviewer and clearly stated that we are working with a hypomorphic condition. The quantification (Fig. S1A) indicates a minimum of 70-75% reduction in MLC phosphorylation and by proxy activity. It is worth noting that this reduction is sufficient to interfere with p-MLC accumulation at the boundary as shown in Fig 7B.

5. For Fig. 5, it is good to see the RLC depletion, but the RLC is not 100% absent. What is the level of reduction, at least at the level of fluorescence differences? The statement is "very effective in

© 2023. Published by The Company of Biologists under the terms of the Creative Commons Attribution License (https://creativecommons.org/licenses/by/4.0/).

depleting" is not especially quantitative. From the images, it is likely around a 70% reduction, which is probably not sufficient to interpret negative results.

Our quantification indicates that the residual p-MLC in cells with sqhRi does not exceed 20% of normal levels and the depletion is more drastic in most cells (Fig. S2B). We have added this information to the text:

Page 7: "The Myosin levels in cell clones expressing *sqhRi* did not exceed 20% of normal levels, with no residual Myosin cables detected at the junctions of these cells (Fig. 5A-A', and S1B)."

6. Regarding statistical analysis, according to the Methods section, "two- sample t-tests" were used. This is incorrect for two reasons. First, many data sets incorporate more than two groups. Second, many distributions appear not to be normally (Gaussian) distributed. Therefore, two-or-more group analyses (e.g. parametric ANOVA or nonparametric Kruskal Wallis) should be used. For nonparametric, Wilcoxon should be used for follow up pair-wise comparisons. Also, box-and-whiskers plots still obscure actual sample distributions. Please use dot plots so that all iterations may be observed.

We thank the reviewer for the suggestion. Indeed, a non-parametric test is more appropriate for our data. We have updated all our statistical analysis using unpaired two-samples Wilcoxon tests. We also show individual data points on our new plots as requested by the reviewer. The new statistical analysis did not change the interpretation for the vast majority of the results, but it did show a slight change for the sqh-RNAi experiments in Figure 5. The old (left) and the new (right) plots are shown below. With the new analysis, the clone rescue provided by sqh- RNAi is mildly less significant (5F, p=0,042 vs old p=0.037), whereas the effect on roundness of the clones is now more significant (5K, p=0,008 instead of former p=0.01). Notably, despite a relaxation at the boundary, cells inside the clone (dLMO + sqhRi) are still constricted, indicated by smaller apical areas (5L). We reflect these findings on our description of the data.

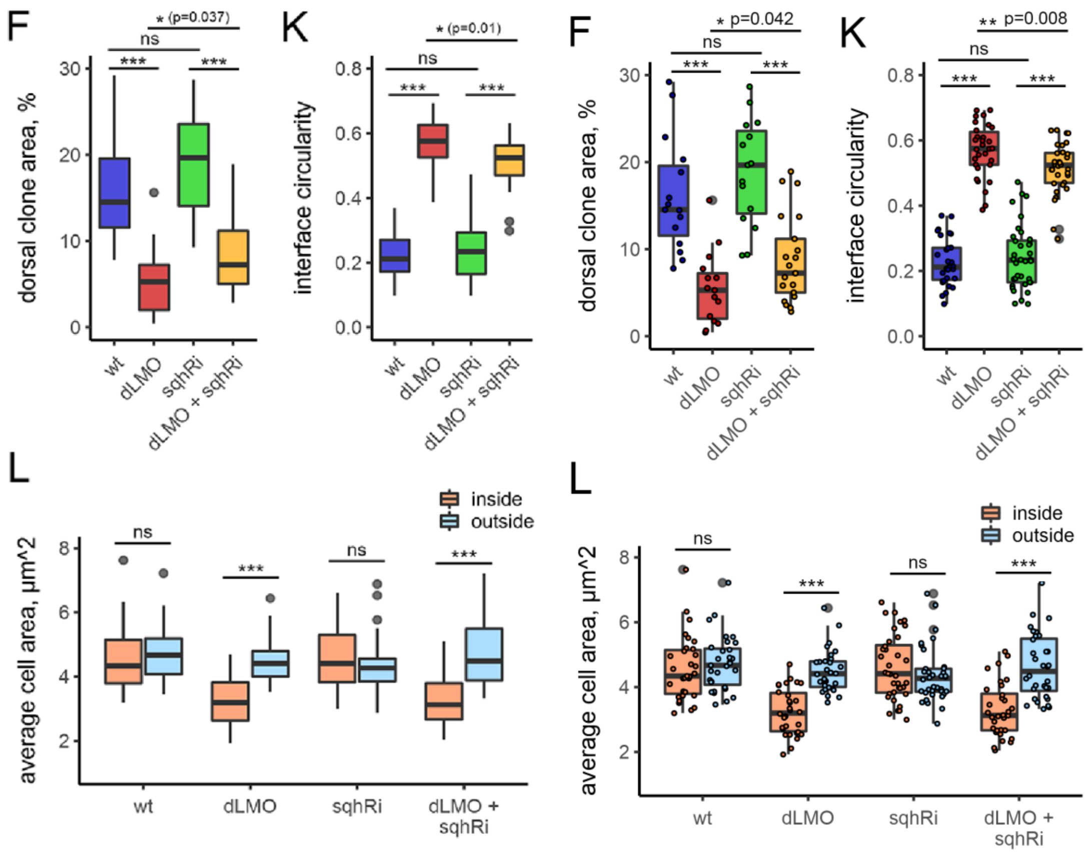

7.Due to the statistical concern in point 6, it is possible that the impact of RLC knockdown (shqRi) is significantly different, even in a WT background.

© 2023. Published by The Company of Biologists under the terms of the Creative Commons Attribution License (https://creativecommons.org/licenses/by/4.0/).

No, use of a non-parametric test did not make the differences significant.

8. The concept of "rounding" solely due to myosin II contractility is not a general idea. Other features like Laplace pressures are well known to drive cell rounding in a myosin II-independent manner. Therefore, one would not necessarily expect myosin II to be the only mechanisms for rounding of cells or groups of cells.

Minor comments:

1.Fig. 3: Arrows should be added to point out specific structures described. One can interpret a lot of things in these images in different ways, and while I think I know what the authors are pointing out, please make it specifically clear.

We have added arrowheads pointing at the cables.

Final Comments:
I think a lot of this can be addressed through re-writing and separating out the experimental observations from interpretations. Then, by incorporating the above considerations, they can provide a more rigorous interpretation in the Discussion. Quantification of the knockdown levels is also essential. My suspicion is that the knockdown scenarios are only ~70-80% effective, which could mean that there is ~20-30% of functional myosin II still in the cells.

The residual level might have ample dynamic range to close the gap given the load dependency of myosin II duty ratio and other force-sharing machinery that is undoubtedly present here.

Reviewer 3 Advance Summary and Potential Significance to Field:
In this manuscript, Hamaratoglu and colleagues utilize the wing primordium of Drosophila to analyze the role of non-muscle Myosin II, known to regulate contractile forces in a cell, in the sorting and elimination of mis-specified cells. The wing primordium is subdivided into a dorsal and a ventral compartment, which will give rise to the two surfaces of the adult wing, and the boundary between these two populations is well known to be maintained by the activity of Myosin II. Clones of cells of dorsal or ventral identify induced in opposite compartments are round, their boundaries are smooth and clones are lost by apoptosis. Here, authors present convincing experimental evidence that Myosin II depletion does not interfere with the loss of these clones and does not rescue the shape and smoothness of these clones. They have carried out these experiments with the use of different tools (UAS-transgenes, mutant alleles) and have manipulated MyoII activity in the different cell populations. These data clearly contribute to our understanding of the molecular mechanisms underlying the removal of misspecified cells in development and open new avenues towards the identification of the real actors.

Reviewer 3 Comments for the Author:
Overall, the ms is beautifully written, the figures are clear, self-explanatory and nicely presented and organized and the discussion summarizes and contextualized in a smart way the results obtained in this study. Thus, my suggestion is a straightforward acceptance of the paper without any further delay. I congratulate authors for this nice piece.

We thank the reviewer for this assessment.

Minor comments:

(1)In the second paragraph of the Introduction, I would suggest to include references in all sentences to acknowledge the work of previous labs.
Done!

(2)In the fourth paragraph of the Introduction, the names of transcription factors should not be in italics and the first letter a capital one).
Thanks for pointing this out, corrected to protein name nomenclature.

(3)Authors might want include the reference of Becam et al 2011 where the role of Myosin II in DV boundary formation is further analyzed.

© 2023. Published by The Company of Biologists under the terms of the Creative Commons Attribution License (https://creativecommons.org/licenses/by/4.0/).

Becam et al was already cited (former ref. #21), but we further added the following sentence (page 3-4):

“Ap induces the expressions of the N ligand Serrate and its modulator Fringe in dorsal cells and restricts Delta expression to the ventral cells ensuring a stripe of N signaling along the D/V boundary (Rauskolb et al., 1999, O'Keefe and Thomas, 2001, Becam and Milán, 2008). This stripe of N ensures boundary maintenance *via* reduced proliferation at the D/V boundary by repression of bantam (Becam et al., 2011) as well as increasing the cell bond tension as a result of accumulations of Myosin II and Enabled, an actin regulator (Michel et al., 2016, Becam et al., 2011).”

Second decision letter

MS ID#: JOCES/2022/259935

MS TITLE: Elimination of Aberrantly Specified Cell Clones Is Independent of Interfacial Myosin II Accumulation

AUTHORS: Olga Klipa, Menna El Gammal, and Fisun Hamaratoglu
ARTICLE TYPE: Research Article

We have now reached a decision on the above manuscript.

To see the reviewers' reports and a copy of this decision letter, please go to: https://submit-jcs.biologists.org and click on the 'Manuscripts with Decisions' queue in the Author Area. (Corresponding author only has access to reviews.)

As you will see, the reviewers gave favourable reports but raised on issue in the summary statement that I agree is important to correct. I hope that you can complete this revision so that I can accept your manuscript.

Please ensure that you clearly highlight all changes made in the revised manuscript. Please avoid using 'Tracked changes' in Word files as these are lost in PDF conversion.

I should be grateful if you would also provide a point-by-point response detailing how you have dealt with the points raised by the reviewers in the 'Response to Reviewers' box. Please attend to all of the reviewers' comments. If you do not agree with any of their criticisms or suggestions please explain clearly why this is so.

Reviewer 1

Advance summary and potential significance to field

The authors have adressed all my concerned thanks to new paragraphs in the introduction and the discussion. I am fully supportive for publication.

Comments for the author

I just have very minor point (requiring text editing):
Right now the summary statement says: "Myosin II accumulation along the boundary between differently fated cells is not sufficient to drive elimination of harmful cells in Drosophila larval wing discs."

© 2023. Published by The Company of Biologists under the terms of the Creative Commons Attribution License (https://creativecommons.org/licenses/by/4.0/).

I am not sure this really reflects the result of the manuscript since "is not sufficient" would suggest that the authors manage to increase MyoII at boundary without fate alteration and show that this does not trigger compaction and death.
I think it would be more appropriate to say that "Myosin II accumulation along the boundary between differently fated cells is not necessary to drive elimination and sorting of harmfull cells"

Reviewer 2

Advance summary and potential significance to field

The authors have adequately addressed the concerns raised.

Comments for the author

The authors have adequately addressed the concerns raised.

Reviewer 3

Advance summary and potential significance to field

The paper should be published.

Comments for the author

Authors hava satisfactorily addressed my minor comments.

Second revision

Author response to reviewers' comments

Dear Reviewers,

Thanks for all the time you invested in evaluating our work!

Best wishes,
Fisun

Third decision letter

MS ID#: JOCES/2022/259935

MS TITLE: Elimination of Aberrantly Specified Cell Clones Is Independent of Interfacial Myosin II Accumulation

AUTHORS: Olga Klipa, Menna El Gammal, and Fisun Hamaratoglu
ARTICLE TYPE: Research Article

I am happy to tell you that your manuscript has been accepted for publication in Journal of Cell Science, pending standard ethics checks.

© 2023. Published by The Company of Biologists under the terms of the Creative Commons Attribution License (https://creativecommons.org/licenses/by/4.0/).
